# A shared genetic contribution to osteoarthritis and COVID-19 outcomes: a large-scale genome-wide cross-trait analysis

**DOI:** 10.3389/fimmu.2023.1184958

**Published:** 2023-06-16

**Authors:** Yi-Xuan Huang, Tian Tian, Ji-Xiang Huang, Jing Wang, Cong Sui, Jing Ni

**Affiliations:** ^1^ Department of Epidemiology and Biostatistics, School of Public Health, Anhui Medical University, Hefei, Anhui, China; ^2^ Department of Endocrinology, The First Affiliated Hospital of Anhui Medical University, Hefei, Anhui, China; ^3^ Department of Orthopedics Trauma, The First Affiliated Hospital of Anhui Medical University, Hefei, Anhui, China

**Keywords:** osteoarthritis, COVID-19 severity, genetic correlation, Mendelian randomization, cross-trait analysis

## Abstract

**Background:**

Patients with osteoarthritis (OA) are exposed to an increased risk of adverse outcomes of COVID-19, and they tend to experience disruption in access to healthcare services and exercise facilities. However, a deep understanding of this comorbidity phenomenon and the underlying genetic architecture of the two diseases is still unclear. In this study, we aimed to untangle the relationship between OA and COVID-19 outcomes by conducting a large-scale genome-wide cross-trait analysis.

**Methods:**

Genetic correlation and causal relationships between OA and COVID-19 outcomes (critical COVID-19, COVID-19 hospitalization, and COVID-19 infection) were estimated by linkage disequilibrium score regression and Mendelian Randomization approaches. We further applied Multi-Trait Analysis of GWAS and colocalization analysis to identify putative functional genes associated with both OA and COVID-19 outcomes.

**Results:**

Significant positive genetic correlations between OA susceptibility and both critical COVID-19 (r_g_=0.266, *P*=0.0097) and COVID-19 hospitalization (r_g_=0.361, *P*=0.0006) were detected. However, there was no evidence to support causal genetic relationships between OA and critical COVID-19 (OR=1.17[1.00-1.36], *P*=0.049) or OA and COVID-19 hospitalization OR=1.08[0.97-1.20], *P*=0.143). These results were robustly consistent after the removal of obesity-related single nucleotide polymorphisms (SNPs). Moreover, we identified a strong association signal located near the *FYCO1* gene (lead SNPs: rs71325101 for critical COVID-19, *P_meta_* =1.02×10^-34^; rs13079478 for COVID-19 hospitalization, *P_meta_* =1.09×10^-25^).

**Conclusion:**

Our findings further confirmed the comorbidity of OA and COVID-19 severity, but indicate a non-causal impact of OA on COVID-19 outcomes. The study offers an instructive perspective that OA patients did not generate negative COVID-19 outcomes during the pandemic in a causal way. Further clinical guidance can be formulated to enhance the quality of self-management in vulnerable OA patients.

## Introduction

The global pandemic of Coronavirus disease (COVID-19) pandemic has been plaguing the world since late 2019. As of 18 August, 2022, the cumulative number of confirmed patients worldwide exceeded 59 million (1). Analysis of epidemiological data showed an increased risk of adverse outcomes of COVID-19 in patients with immune-mediated arthritis (2–4). In fact, COVID-19 also has a significant impact on the most prevalent type of chronic arthritis, osteoarthritis (OA) (5), which is among the most important public health problems worldwide, with more than 300 million people currently affected by the condition (6).

Observational studies have reported that patients with OA are likely to be infected by COVID-19 and that the condition may be aggravated (7, 8). To avoid infection during pandemics, some patients with OA have had to endure pain and reduce the frequency at which they see doctors, undergo operations, or apply non-steroidal anti-inflammatory drugs (NSAIDs) (9). However, Wang et al. (10) reported no apparent associations between OA and the risk of COVID-19. Additionally, according to a UK cohort study, no increased risk of COVID-19-related adverse outcomes was observed among OA patients who were prescribed NSAIDs. A deeper understanding of the phenomenon of comorbidity in patients with COVID-19 and OA is warranted to offer effective clinical instruction and improve the quality of self-management in this vulnerable population.

There is growing evidence that COVID-19 traits often share highly polygenic genetic components with several complex diseases, such as idiopathic pulmonary fibrosis (11), type 2 diabetes (12), and asthma (13). Although there have been recent reports that some genetic markers of OA are associated with COVID-19 outcomes, the underlying genetic basis of the relationship between these diseases has not been thoroughly assessed (14–16). Here, we systematically estimate the shared genetic architecture of OA and three COVID-19 traits, including critical COVID-19, COVID-19 hospitalization, and COVID-19 infection, as well as further assessing the causality of OA and COVID-19 traits using a Mendelian randomization (MR) approach.

## Methods

### Study design and population

**Figure 1:**
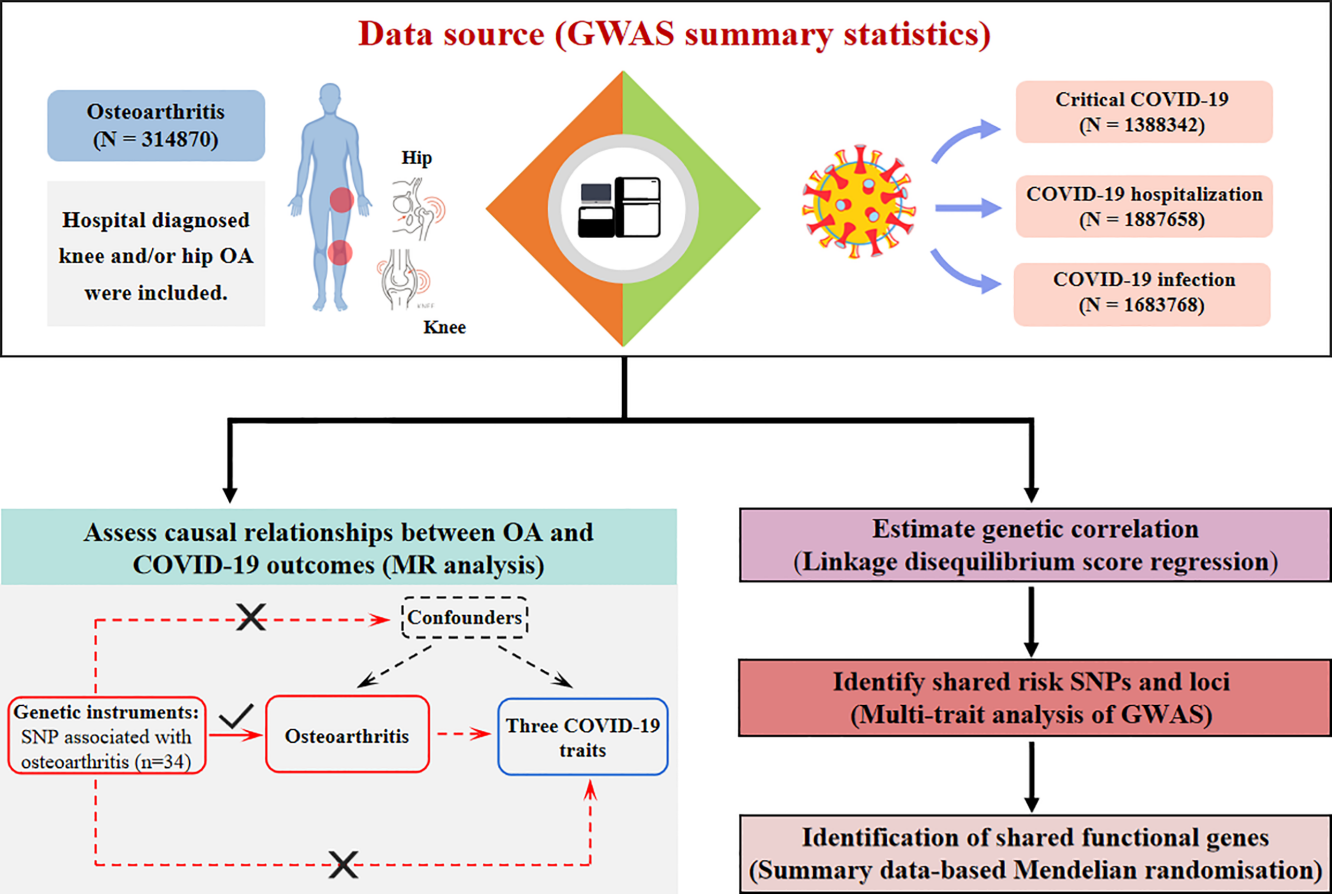
Flowchart of the current study. OA patients were contained with a sample size of 314870. Three COVID-19-related traits include critical COVID-19, COVID-19 hospitalization, and COVID-19 infection, and their sample sizes are 1388342, 1887658, and 1683768 respectively. Study approaches consist of assessing causal relationships using Mendelian Randomization, and identifying genetic correlations, shared risk SNPs, and functional genes using LDSC, MTAG, and SMR analysis. OA, osteoarthritis; SNP: single nucleotide polymorphism; LDSC, linkage disequilibrium score regression; MTAG, multi-trait analysis of GWAS; SMR, summary data-based Mendelian randomization.

A brief flowchart of the current study is shown in **Figure 1**. GWAS summary level data for COVID-19 were obtained from the 5^th^ edition data (release date: 18 January, 2021) of the COVID-19 host genetics consortium (17). To minimize the bias introduced by population stratification, participants were restricted to those of European descent. Three COVID-19-related traits were characterized as follows: 1. critical COVID-19, defined as COVID-19-confirmed individuals with very severe respiratory symptoms or those who died from the disease (up to 5101 cases and 1,383,241 controls); 2. COVID-19 hospitalization, (up to 9986 cases and 1,877,672 controls); and 3. SARS-CoV2 infection (up to 38,984 cases and 1,644,784 controls). We obtained the largest GWAS summary data for hospital-diagnosed OA susceptibility from a GWAS meta-analysis comprising a sample size of 314,870 individuals of European ancestry (18). Hospital-diagnosed OA was defined as individuals with an ICD-10 and/or ICD-9 hospital record code captured from the hospital episode statistic (HES) for OA at any site (18). Summary-level data for rheumatoid arthritis (RA) were extracted from the most extensive GWAS meta-analysis of European ancestry, comprising a total of 58,284 individuals (14,361 cases and 43,923 controls) (19). Details of the data sources used in the current study are summarized in **Supplementary Table 1**.

### Linkage disequilibrium score regression

Linkage disequilibrum score regression (LDSC) software (https://github.com/bulik/ldsc) was used to estimate the single nucleotide polymorphism (SNP)-based heritability of each trait and genetic correlations between OA and COVID-19 outcomes, based on GWAS summary statistics (20). SNPs within the major histocompatibility complex (MHC) region were removed due to the complex structure of linkage disequilibrium (LD) structure in the region. The 1000 Genomes Project European LD score reference panel was adopted throughout the analyses. As obesity is a recognized independent risk factor for both arthritis (21) and COVID-19 (22), we performed further LDSC analysis to estimate the genetic correlation between RA and COVID-19 outcomes. A Bonferroni-corrected *P*-value < 0.017 (0.05/3 = 0.017) was set as the threshold for significance in the LDSC analysis.

### Multi-trait analysis of GWAS

A generalized inverse-variance-weighted meta-analysis was conducted using Multi-trait analysis of GWAS (MTAG) to identify risk SNPs associated with joint phenotypes of OA and each of the analyzed COVID-19-related traits. This approach enabled combined analysis of multiple traits and thus boosted the statistical power to detect genetic associations for each trait (23). A genome-wide significance level of *P* < 5 × 10^-8^ was set for MTAG.

### Colocalization analysis

Summary data-based Mendelian Randomization (SMR) and Heterogeneity in Dependent Instrument (HEIDI) methods were applied to identify putative pleiotropic genes underlying OA and each of the critical-COVID-19 and COVID-19 hospitalization traits, by jointly analyzing the results of the cross-trait meta-analysis and the publicly available cis-eQTL summary data from whole blood and lung tissue of the Genotype-tissue expression (GTEx) project (24). Significant SMR associations were defined if they passed the FDR correction (*P*_FDR_ < 0.05) and also surpassed the HEIDI-outlier test (*P*_HEIDI_ > 0.05).

### MR analysis

A two-sample MR analysis was conducted to examine whether the relationships between OA and COVID-19 outcomes were causal. Instrumental variants (IVs) for OA were required to meet three criteria, including that the markers: 1. were strongly associated with OA; 2. affected COVID-19 infection only through their effect on OA, and, 3. most importantly, were independent of any confounding variables of associations between OA and COVID-19-related traits. LD clumping (r^2^<0.001, 10000kb) was used to select independent SNPs. Methods used for MR analysis included inverse variance weighted (IVW) (25), weighted median (26), weighted mode (27), and MR-Egger (28), followed by pleiotropy test and leave-one-out analysis. Phenoscanner v2 (29) was applied to check whether any of the selected IVs for OA were associated with obesity-related phenotypes (*P*<1×10^-5^). A sensitivity analysis was conducted by removing all obesity-related SNPs. All statistical analyses were performed in R (version 4.1.3) using the packages MendelianRandomization (version 0.5.6) and MRPRESSO (version 1.0). A Bonferroni-corrected threshold of *P*=0.017 was considered significant for MR analyses.

## Results

### Genetic correlation of OA with COVID-19 outcomes

As shown in **Table 1**, the liability-scale SNP heritability values were 8.92% for OA, 0.35% for critical COVID-19, 0.19% for COVID-19 hospitalization, and 0.13% for COVID-19 infection, respectively. We found significant positive genetic correlations between OA susceptibility and critical COVID-19 (r_g_=0.266, *P*=0.0097), as well as with COVID-19 hospitalization (r_g_=0.361, *P*=0.0006). A positive genetic correlation was also detected between OA susceptibility and COVID-19 infection, although it did not achieve the Bonferroni-corrected significance threshold (r_g_=0.280, *P*=0.0238). Moreover, no genetic correlations were detected between RA susceptibility and COVID-19 outcomes (**Supplementary Table 2**).

**Table 1:** Genetic correlations between osteoarthritis and COVID-19.

Phenotype	SNP-heritability (SE)	Genetic correlation with osteoarthritis	
		r_g_ (SE)	P-value
Osteoarthritis	8.92% (0.0114)	–	–
Critical COVID-19	0.35% (0.0007)	0.266 (0.103)	9.70E-03
COVID-19 hospitalization	0.19% (0.0005)	0.361 (0.106)	6.00E-04
COVID-19 infection	0.13% (0.0003)	0.280 (0.124)	0.0238

SNP, single nucleotide polymorphism; SE, standard error.

### Multi-trait analysis of OA and COVID-19 severity

We performed MTAG to conduct a cross-trait meta-analysis to detect the loci that were significantly associated with both OA and COVID-19 severity. We identified 357 shared genetic loci associated with both critical COVID-19 and OA and 288 associated with both COVID-19 hospitalization and OA (*P*_meta_<5×10^-8^) (**Supplementary Tables 3, 4** **)**. After excluding loci with an inconsistent direction of effect, shared signals for COVID-19 severity and OA were mainly mapped to chromosomes 3, 19, and 21. The strongest association signals for the two COVID-19 traits with OA mapped close to the *FYCO1* gene (lead SNP: rs71325101 for critical COVID-19, *P*_meta_=1.02×10^-34^; rs13079478 for COVID-19 hospitalization, *P*_meta_=1.09×10^-25^).

### Colocalization analysis

By conducting SMR and HEIDI methods, several non-MHC region SNPs were identified as common shared genetic loci including rs143334143 (*TCF19*), rs2277732 (*DPP9*), rs77534576 (*DLX3*), and rs13081151 (*FLT1P1*), among others, which were significantly associated with OA-critical COVID-19 combined trait. Rs2277732 (*DPP9*), rs13081151 (*FLT1P1*), and rs11085727 (*TYK2*), among others, were considered significantly associated3with OA-COVID-19 hospitalization combined trait (**Supplementary Table 5**).

### MR analysis

After LD clumping, 34 independent IVs were selected for OA (**Supplementary Table 6**). Based on the IVW method, there was no evidence to support significant causal genetic relationships between OA and critical COVID-19 (OR=1.17[1.00-1.36], *P*=0.049), OA and COVID-19 hospitalization (OR=1.08[0.97-1.20], *P*=0.143), or OA and COVID-19 infection (OR=1.06[1.00-1.11], *P*=0.034). Using the MR Egger method, we found that a genetically predicted OA was positively correlated with a higher risk of suffering from critical COVID-19 (OR=1.72[1.18-2.51], *P*=0.009) (**Table 2**). In addition, our analysis suggested no significant evidence of horizontal pleiotropy. The direction and precision of the summary association between OA and COVID-19 remained largely unchanged using a leave-one-out approach (**Supplementary Figure 1**). Among the selected SNPs of OA, four of which were associated with obesity phenotype at a significant level of *P*<1×10^-5^ (rs2820436, rs73080980, rs6977416, and rs143383) (**Supplementary Table 7**). The results were robust after removing four obesity-related SNPs (**Supplementary Table 8** and **Supplementary Figure 2** **).**

**Table 2:** Results of Mendelian randomization analyses evaluating causal relationships between osteoarthritis and COVID-19 outcomes.

Outcomes	n.SNPs	Methods	OR	95% CI	*P* value
Critical COVID-19	28	Inverse variance weighted	1.17	1.00, 1.36	0.049
		MR Egger	1.72	1.18, 2.51	0.009
		Weighted median	1.18	0.95, 1.46	0.143
		Weighted mode	1.46	0.97, 2.19	0.081
COVID-19 hospitalization	26	Inverse variance weighted	1.08	0.97, 1.20	0.143
		MR Egger	1.16	0.88, 1.53	0.298
		Weighted median	1.08	0.94, 1.23	0.281
		Weighted mode	1.05	0.82, 1.34	0.713
COVID-19 infection	26	Inverse variance weighted	1.06	1.00, 1.11	0.034
		MR Egger	1.09	0.95, 1.24	0.250
		Weighted median	1.03	0.96, 1.11	0.366
		Weighted mode	1.03	0.90, 1.17	0.716

n.SNPs, number of single nucleotide polymorphisms; OR, odds ratio; CI, confidence interval.

## Discussion

Our study was the first to decipher in-depth the genetic architecture underlying the relationships between OA and three COVID-19 traits. Leveraging large-scale GWAS summary statistics data, we identified positive genetic correlations and shared genetic loci, genes between OA and COVID-19 traits. Further, MR analysis did not support that OA increased the risk of COVID-19 susceptibility and severity.

Our findings support a shared genetic contribution to OA and COVID-19 outcomes and indicate that the relationships are likely to be comorbid, rather than causal. COVID-19 is devastatingly deleterious to the human body by causing an aggressive immune response designated as a cytokine storm, which leads to multi-organ failure and finally death (30). The pro-inflammation effects of various cytokines are also well-established factors contributing to the pathophysiology of OA (31). It an plays important role in OA progression by stimulating matrix metalloproteinase (MMPs) development, thereby leading to matrix degradation (32). Recently, there have been reports that mesenchymal stem/stromal cells (MSCs) secrete immunomodulatory cytokines such as TGFBI (33), PGE2, and IL-6, which are expected to be promising therapeutic targets for both OA and critical COVID-19 (34), strongly supporting shared features in the etiological pathways leading to these two conditions. Obesity is an established risk factor for both arthritis and COVID-19 (35–37). To eliminate the effect of obesity, we further estimated the genetic correlation between RA and COVID-19 and did not detect any genetic correlations between RA susceptibility and COVID-19 outcomes. In the primary IVW MR analysis, genetically predicted OA was not associated with any of the COVID-19 outcomes. Although a significant causal effect of OA on critical COVID-19 was detected using the MR-Egger method, this approach is relatively imprecise compared with the IVW analysis (38). These results were robustly consistent after the removal of obesity-related SNPs. Diseases such as depression and diabetes are also risk factors for OA and COVID-19 according to recent studies (39–42). Therefore, to control the confounders we have additionally searched whether any of the selected IVs for OA were associated with depression and diabetes-related phenotypes (*P*<1×10^-5^) in Phenoscanner v2. We did not find any IVs which are correlated with depression or diabetes.

*FYCO1* gene was observed to be strongly associated with two COVID-19 traits and OA. It encodes a RAB7 adapter protein implicated in the microtubule transport of autophagosomes (43). Previous GWAS studies have also reported an association signal with COVID-19 at the 3p21.31 locus, which contains a cluster of genes including *FYCO1* (15, 17). We also identified several putative functional genes as associated with both diseases, including *DLX3*, *DPP9*, *TCF19*, and *TYK2*. Distal-less (*DLX*) family genes play crucial roles in bone tissue development and regulate osteoblast differentiation (44). A recent study revealed that the osteogenic differentiation of human bone marrow MSCs is enhanced by *DLX3* overexpression through the Wnt/β-catenin pathway (45). On the other hand, the outcomes of COVID-19 patients were substantially improved by MSCs treatment in experimental studies and this approach is expected to be applied to patients with COVID-19 in the clinic (46, 47). These reports are consistent with our results showing that OA patients are not causally susceptible to COVID-19 infection. *DPP9* encodes a serine protease with a key role in inflammasome activation (48). On the contrary, *TYK2* is thought to be one of the four gene targets for JAK inhibitors and the anti-inflammatory properties of which have nowadays been enthusiastically discussed. Symptoms of gastrointestinal inflammation or psoriasis are frequently observed in patients with critical COVID-19 and OA (49, 50), indicating comorbid properties among those diseases, and the genes discussed above are likely to be future therapeutic targets.

We acknowledge several limitations of the current study. First, the available summary statistics used in our MR analysis consist solely of data from a population with European ancestry and therefore are not typical of the overall population. Second, data were not organized by age or sex, precluding the possibility of further analysis of the relationship between the two diseases in stratified populations. Finally, despite the fact we explicitly explored the underlying genetic architecture of OA and COVID-19, the specific mechanisms involved remain unclear and further analysis on molecules and pathways is warranted.

## Conclusion

In summary, our study provides innovative insights into the genetic architecture underlying the relationship between OA and three COVID-19 traits. We identified positive genetic correlations and putative shared functional genes between them. However, OA did not increase the risk of COVID-19 susceptibility or severity. Therefore, the cancellation or delay in elective joint replacement surgeries and the reduction in physical activities were not compulsory. Our study is of great significance to the transition of management and clinical guidance of OA patients during the COVID-19 pandemic.

## Data availability statement

The original contributions presented in the study are included in the article/**Supplementary material**. Further inquiries can be directed to the corresponding authors.

## Author contributions

JN and CS take responsibility for the content of the manuscript, including the conception and design of the study and final approval of the version to be submitted. Y-XH and TT contributed to the acquisition, analysis, and interpretation of the data and wrote the manuscript. J-XH and JW contributed to the analysis and interpretation of the data. All authors contributed to the article and approved the submitted version.
