# Supplementary material for: A shared genetic contribution to osteoarthritis and COVID-19 outcomes: a large-scale genome-wide cross-trait analysis
Source: Front Immunol. 2023 Jun 16;14:1184958. doi: 10.3389/fimmu.2023.1184958 (PMC10311546; doi:10.3389/fimmu.2023.1184958)
Supplement: Supplementary file 1 [file DataSheet_1.docx]

**Table S1.** Details of the data sources used in current study.

| **Phenotype** | **Sample size** | **Source** |
| --- | --- | --- |
| Osteoarthritis | 314870 | https://www.ebi.ac.uk/gwas/publications/29559693 |
| Rheumatoid arthritis | 58284 | http://plaza.umin.ac.jp/~yokada/datasource/software.htm |
| Critical COVID-19 | 1388342 | https://www.covid19hg.org/results/r5/ |
| COVID-19 hospitalization | 1887658 | https://www.covid19hg.org/results/r5/ |
| COVID-19 infection | 1683768 | https://www.covid19hg.org/results/r5/ |

**Table S2.** Genetic correlations between rheumatoid arthritis and COVID-19 severity.

| **Phenotype** | **Genetic correlation with rheumatoid arthritis** | |
| --- | --- | --- |
|  | r_g_ (SE) | P value |
| Rheumatoid arthritis | **-** | **-** |
| Critical COVID-19 | 0.1251 (0.0777) | 0.1075 |
| COVID-19 hospitalization | 0.1014 (0.0755) | 0.1794 |
| COVID-19 infection | 0.0867 (0.0758) | 0.2524 |
| SNP, single nucleotide polymorphism; SE, standard error. | | |

**Table S3.** Significant genetic variants associated with cross-trait osteoarthritis and critical COVID-19.

| **SNP** | **Chr** | **Position** | **A1** | **A2** | **Beta_mtag_** | **SE_meta_** | *P*_meta_ |  | **Osteoarthritis** | | |  | **Critical COVID-19** | | |
| --- | --- | --- | --- | --- | --- | --- | --- | --- | --- | --- | --- | --- | --- | --- | --- |
|  |  |  |  |  |  |  |  |  | **Beta** | **SE** | ***P*_value** |  | **Beta** | **SE** | ***P*_value** |
| rs6675468 | 1 | 155040654 | T | C | -0.014 | 0.002 | 1.82E-08 |  | -0.062 | 0.026 | 1.81E-02 |  | -0.167 | 0.032 | 1.84E-07 |
| rs10510748 | 3 | 46178538 | G | A | 0.017 | 0.002 | 1.41E-14 |  | 0.031 | 0.029 | 2.89E-01 |  | 0.232 | 0.03 | 2.05E-14 |
| rs10510749 | 3 | 46180416 | T | C | 0.017 | 0.002 | 2.71E-14 |  | 0.024 | 0.03 | 4.18E-01 |  | 0.239 | 0.031 | 2.60E-14 |
| rs10510750 | 3 | 46274886 | C | T | 0.017 | 0.002 | 4.32E-13 |  | 0.018 | 0.032 | 5.83E-01 |  | 0.246 | 0.034 | 2.89E-13 |
| rs114115904 | 3 | 46268664 | T | C | 0.019 | 0.002 | 2.98E-15 |  | 0.006 | 0.032 | 8.52E-01 |  | 0.265 | 0.033 | 8.89E-16 |
| rs115102354 | 3 | 46222037 | G | A | 0.025 | 0.003 | 4.68E-20 |  | 0.020 | 0.038 | 5.99E-01 |  | 0.359 | 0.039 | 1.75E-20 |
| rs116055491 | 3 | 46573675 | G | A | 0.022 | 0.004 | 2.81E-08 |  | 0.090 | 0.059 | 1.32E-01 |  | 0.325 | 0.061 | 8.65E-08 |
| rs11919943 | 3 | 46266726 | C | T | 0.018 | 0.002 | 2.93E-14 |  | 0.014 | 0.031 | 6.58E-01 |  | 0.238 | 0.031 | 1.50E-14 |
| rs11923627 | 3 | 46301786 | G | A | 0.016 | 0.002 | 3.77E-12 |  | 0.042 | 0.031 | 1.83E-01 |  | 0.219 | 0.032 | 9.15E-12 |
| rs11926063 | 3 | 46263717 | G | A | 0.017 | 0.002 | 4.56E-14 |  | 0.012 | 0.031 | 6.97E-01 |  | 0.238 | 0.031 | 2.17E-14 |
| rs11929489 | 3 | 46259915 | A | C | 0.016 | 0.002 | 9.05E-13 |  | 0.023 | 0.029 | 4.41E-01 |  | 0.213 | 0.03 | 8.85E-13 |
| rs12108042 | 3 | 46086083 | G | A | 0.020 | 0.002 | 8.62E-27 |  | 0.001 | 0.025 | 9.83E-01 |  | 0.277 | 0.025 | 5.34E-28 |
| rs12639314 | 3 | 46141504 | C | A | 0.011 | 0.002 | 1.15E-10 |  | 0.007 | 0.022 | 7.53E-01 |  | 0.168 | 0.026 | 6.45E-11 |
| rs13059906 | 3 | 46208858 | C | T | 0.016 | 0.002 | 2.52E-13 |  | 0.032 | 0.03 | 2.87E-01 |  | 0.222 | 0.031 | 3.87E-13 |
| rs13060713 | 3 | 46272709 | C | A | 0.019 | 0.002 | 4.25E-15 |  | 0.006 | 0.032 | 8.48E-01 |  | 0.265 | 0.033 | 1.30E-15 |
| rs13061548 | 3 | 46226646 | C | T | 0.017 | 0.002 | 7.78E-13 |  | 0.034 | 0.029 | 2.46E-01 |  | 0.212 | 0.03 | 1.25E-12 |
| rs13062450 | 3 | 46227171 | G | T | 0.017 | 0.002 | 2.32E-14 |  | 0.028 | 0.029 | 3.43E-01 |  | 0.231 | 0.03 | 2.80E-14 |
| rs13065041 | 3 | 46289403 | T | G | 0.017 | 0.002 | 5.89E-13 |  | 0.027 | 0.032 | 4.05E-01 |  | 0.246 | 0.034 | 6.32E-13 |
| rs13065351 | 3 | 46327512 | G | T | 0.018 | 0.003 | 5.34E-12 |  | 0.029 | 0.032 | 3.67E-01 |  | 0.238 | 0.035 | 5.69E-12 |
| rs13066062 | 3 | 46018344 | A | G | 0.024 | 0.002 | 7.27E-33 |  | 0.007 | 0.027 | 7.88E-01 |  | 0.334 | 0.027 | 4.49E-34 |
| rs13066516 | 3 | 45975443 | T | C | 0.024 | 0.002 | 4.13E-33 |  | 0.006 | 0.027 | 8.30E-01 |  | 0.337 | 0.028 | 2.15E-34 |
| rs13067058 | 3 | 46273766 | A | G | 0.018 | 0.002 | 5.61E-14 |  | 0.008 | 0.032 | 7.97E-01 |  | 0.257 | 0.034 | 2.14E-14 |
| rs13068145 | 3 | 46332276 | A | C | 0.017 | 0.002 | 1.86E-12 |  | 0.029 | 0.032 | 3.69E-01 |  | 0.240 | 0.034 | 2.27E-12 |
| rs13068271 | 3 | 46332184 | A | G | 0.017 | 0.002 | 1.90E-12 |  | 0.029 | 0.032 | 3.66E-01 |  | 0.240 | 0.034 | 2.35E-12 |
| rs13069079 | 3 | 46000870 | A | G | 0.024 | 0.002 | 1.30E-32 |  | 0.004 | 0.027 | 8.70E-01 |  | 0.334 | 0.027 | 6.18E-34 |
| rs13069750 | 3 | 46224850 | T | C | 0.017 | 0.002 | 3.09E-14 |  | 0.023 | 0.030 | 4.40E-01 |  | 0.239 | 0.031 | 2.79E-14 |
| rs13069845 | 3 | 46232132 | T | C | 0.017 | 0.002 | 2.30E-14 |  | 0.024 | 0.030 | 4.20E-01 |  | 0.240 | 0.031 | 2.18E-14 |
| rs13070099 | 3 | 46253214 | C | T | 0.016 | 0.002 | 1.96E-13 |  | 0.018 | 0.030 | 5.45E-01 |  | 0.234 | 0.032 | 1.40E-13 |
| rs13071283 | 3 | 46010007 | C | T | 0.024 | 0.002 | 1.33E-34 |  | 0.014 | 0.026 | 5.87E-01 |  | 0.336 | 0.027 | 1.48E-35 |
| rs13071469 | 3 | 46328627 | C | T | 0.017 | 0.002 | 1.39E-12 |  | 0.029 | 0.032 | 3.73E-01 |  | 0.241 | 0.034 | 1.67E-12 |
| rs13073976 | 3 | 46286663 | C | T | 0.017 | 0.002 | 3.88E-13 |  | 0.027 | 0.032 | 4.06E-01 |  | 0.246 | 0.034 | 4.10E-13 |
| rs13075270 | 3 | 46253789 | C | T | 0.016 | 0.002 | 2.22E-13 |  | 0.025 | 0.029 | 3.87E-01 |  | 0.219 | 0.03 | 2.45E-13 |
| rs13075758 | 3 | 46025048 | A | G | 0.024 | 0.002 | 7.52E-33 |  | 0.008 | 0.027 | 7.77E-01 |  | 0.334 | 0.027 | 4.84E-34 |
| rs13075836 | 3 | 46186638 | C | T | 0.017 | 0.002 | 3.38E-14 |  | 0.024 | 0.030 | 4.26E-01 |  | 0.239 | 0.031 | 3.19E-14 |
| rs13077302 | 3 | 46329475 | C | T | 0.017 | 0.002 | 1.34E-12 |  | 0.029 | 0.032 | 3.67E-01 |  | 0.242 | 0.034 | 1.63E-12 |
| rs13078564 | 3 | 46330205 | C | T | 0.017 | 0.002 | 1.55E-12 |  | 0.026 | 0.032 | 4.20E-01 |  | 0.242 | 0.034 | 1.63E-12 |
| rs13078739 | 3 | 46007488 | A | G | 0.024 | 0.002 | 3.79E-34 |  | 0.011 | 0.027 | 6.72E-01 |  | 0.334 | 0.027 | 3.15E-35 |
| rs13079478 | 3 | 46007823 | T | G | 0.024 | 0.002 | 2.45E-33 |  | 0.005 | 0.027 | 8.54E-01 |  | 0.337 | 0.027 | 1.14E-34 |
| rs13079869 | 3 | 46008087 | A | G | 0.024 | 0.002 | 3.82E-33 |  | 0.008 | 0.027 | 7.69E-01 |  | 0.335 | 0.027 | 2.46E-34 |
| rs13080979 | 3 | 46233690 | G | A | 0.017 | 0.002 | 2.60E-14 |  | 0.024 | 0.030 | 4.20E-01 |  | 0.239 | 0.031 | 2.48E-14 |
| rs13081151 | 3 | 46055716 | A | G | 0.024 | 0.002 | 2.56E-32 |  | 0.007 | 0.027 | 7.93E-01 |  | 0.337 | 0.028 | 1.64E-33 |
| rs13082995 | 3 | 46242477 | C | T | 0.016 | 0.002 | 3.76E-14 |  | 0.027 | 0.029 | 3.52E-01 |  | 0.230 | 0.030 | 4.46E-14 |
| rs13083914 | 3 | 46184680 | T | C | 0.017 | 0.002 | 1.22E-13 |  | 0.023 | 0.030 | 4.37E-01 |  | 0.233 | 0.031 | 1.15E-13 |
| rs13086063 | 3 | 46200860 | C | T | 0.016 | 0.002 | 2.53E-13 |  | 0.032 | 0.029 | 2.78E-01 |  | 0.221 | 0.030 | 4.02E-13 |
| rs13088766 | 3 | 46258226 | A | G | 0.016 | 0.002 | 6.98E-13 |  | 0.025 | 0.029 | 3.98E-01 |  | 0.214 | 0.030 | 7.68E-13 |
| rs13089543 | 3 | 46183560 | G | T | 0.017 | 0.002 | 9.74E-13 |  | 0.029 | 0.029 | 3.22E-01 |  | 0.218 | 0.031 | 1.23E-12 |
| rs13089544 | 3 | 46183561 | C | T | 0.016 | 0.002 | 1.71E-13 |  | 0.029 | 0.029 | 3.22E-01 |  | 0.223 | 0.030 | 2.31E-13 |
| rs13089676 | 3 | 46192785 | T | C | 0.013 | 0.002 | 5.18E-09 |  | 0.029 | 0.030 | 3.35E-01 |  | 0.211 | 0.036 | 7.90E-09 |
| rs13089907 | 3 | 46215710 | T | C | 0.016 | 0.002 | 1.77E-13 |  | 0.032 | 0.03 | 2.81E-01 |  | 0.224 | 0.031 | 2.77E-13 |
| rs13092160 | 3 | 46254791 | C | T | 0.017 | 0.002 | 5.57E-14 |  | 0.020 | 0.03 | 5.10E-01 |  | 0.235 | 0.031 | 4.21E-14 |
| rs13093063 | 3 | 46329368 | T | C | 0.017 | 0.002 | 1.59E-12 |  | 0.029 | 0.032 | 3.67E-01 |  | 0.241 | 0.034 | 1.94E-12 |
| rs13093179 | 3 | 46146314 | T | G | 0.018 | 0.002 | 4.24E-19 |  | 0.000 | 0.027 | 9.95E-01 |  | 0.282 | 0.031 | 5.86E-20 |
| rs13095940 | 3 | 46246816 | G | A | 0.016 | 0.002 | 1.71E-13 |  | 0.020 | 0.030 | 4.97E-01 |  | 0.234 | 0.032 | 1.38E-13 |
| rs13095946 | 3 | 46253812 | A | G | 0.016 | 0.002 | 2.39E-13 |  | 0.019 | 0.030 | 5.26E-01 |  | 0.233 | 0.032 | 1.81E-13 |
| rs13096307 | 3 | 46246953 | T | C | 0.016 | 0.002 | 1.69E-13 |  | 0.020 | 0.030 | 4.97E-01 |  | 0.234 | 0.032 | 1.37E-13 |
| rs13096325 | 3 | 46186838 | G | A | 0.017 | 0.002 | 3.49E-14 |  | 0.024 | 0.030 | 4.30E-01 |  | 0.238 | 0.031 | 3.25E-14 |
| rs13096808 | 3 | 46274906 | T | C | 0.017 | 0.002 | 5.38E-13 |  | 0.017 | 0.032 | 6.05E-01 |  | 0.247 | 0.034 | 3.45E-13 |
| rs13096905 | 3 | 46274766 | A | G | 0.017 | 0.002 | 4.99E-13 |  | 0.017 | 0.032 | 6.05E-01 |  | 0.247 | 0.034 | 3.19E-13 |
| rs13097340 | 3 | 46187118 | A | G | 0.017 | 0.002 | 3.62E-14 |  | 0.024 | 0.030 | 4.30E-01 |  | 0.238 | 0.031 | 3.38E-14 |
| rs13097666 | 3 | 46234467 | T | C | 0.017 | 0.002 | 3.43E-14 |  | 0.024 | 0.030 | 4.15E-01 |  | 0.238 | 0.031 | 3.35E-14 |
| rs13098366 | 3 | 46116787 | A | G | 0.015 | 0.002 | 3.29E-14 |  | 0.000 | 0.023 | 9.88E-01 |  | 0.221 | 0.028 | 5.25E-15 |
| rs13098911 | 3 | 46235201 | T | C | 0.017 | 0.002 | 1.84E-14 |  | 0.025 | 0.030 | 3.98E-01 |  | 0.240 | 0.031 | 1.85E-14 |
| rs13325552 | 3 | 46298377 | T | C | 0.013 | 0.002 | 4.51E-08 |  | 0.031 | 0.032 | 3.39E-01 |  | 0.205 | 0.038 | 7.01E-08 |
| rs13325613 | 3 | 46298373 | T | G | 0.017 | 0.002 | 1.10E-12 |  | 0.039 | 0.032 | 2.25E-01 |  | 0.233 | 0.033 | 2.19E-12 |
| rs13433997 | 3 | 46049765 | C | T | 0.024 | 0.002 | 1.49E-33 |  | 0.020 | 0.027 | 4.48E-01 |  | 0.327 | 0.027 | 3.26E-34 |
| rs138754956 | 3 | 46382462 | A | G | 0.016 | 0.002 | 4.21E-12 |  | 0.035 | 0.032 | 2.76E-01 |  | 0.236 | 0.034 | 7.04E-12 |
| rs138940209 | 3 | 46371581 | A | G | 0.017 | 0.002 | 2.70E-12 |  | 0.039 | 0.032 | 2.28E-01 |  | 0.237 | 0.034 | 5.39E-12 |
| rs1392290 | 3 | 46069210 | A | G | 0.008 | 0.001 | 9.41E-10 |  | 0.000 | 0.017 | 9.95E-01 |  | 0.120 | 0.019 | 3.71E-10 |
| rs140295517 | 3 | 46368690 | G | A | 0.016 | 0.002 | 4.03E-12 |  | 0.034 | 0.032 | 2.87E-01 |  | 0.236 | 0.034 | 6.47E-12 |
| rs140610938 | 3 | 46375555 | G | A | 0.018 | 0.003 | 8.53E-12 |  | 0.037 | 0.032 | 2.50E-01 |  | 0.234 | 0.035 | 1.43E-11 |
| rs142039684 | 3 | 46371793 | C | T | 0.016 | 0.002 | 3.15E-12 |  | 0.035 | 0.032 | 2.79E-01 |  | 0.237 | 0.034 | 5.19E-12 |
| rs142406049 | 3 | 46190155 | A | G | 0.013 | 0.002 | 8.72E-09 |  | 0.026 | 0.030 | 3.77E-01 |  | 0.206 | 0.036 | 1.21E-08 |
| rs146375688 | 3 | 46388700 | T | C | 0.023 | 0.003 | 1.02E-16 |  | 0.043 | 0.039 | 2.71E-01 |  | 0.336 | 0.041 | 1.45E-16 |
| rs1491951 | 3 | 46141844 | A | G | 0.022 | 0.002 | 2.91E-29 |  | 0.002 | 0.027 | 9.44E-01 |  | 0.313 | 0.027 | 1.56E-30 |
| rs150391471 | 3 | 46217740 | A | G | 0.013 | 0.002 | 1.25E-08 |  | 0.024 | 0.030 | 4.17E-01 |  | 0.207 | 0.037 | 1.59E-08 |
| rs1532071 | 3 | 46001063 | A | G | 0.007 | 0.001 | 7.93E-09 |  | 0.024 | 0.016 | 1.27E-01 |  | 0.102 | 0.018 | 2.50E-08 |
| rs1542755 | 3 | 46272440 | T | G | 0.019 | 0.002 | 4.01E-15 |  | 0.008 | 0.032 | 8.12E-01 |  | 0.265 | 0.033 | 1.32E-15 |
| rs1542756 | 3 | 46272162 | T | G | 0.019 | 0.002 | 4.42E-16 |  | 0.016 | 0.032 | 6.14E-01 |  | 0.268 | 0.033 | 2.19E-16 |
| rs1601867 | 3 | 46002962 | C | T | 0.007 | 0.001 | 7.83E-09 |  | 0.024 | 0.016 | 1.36E-01 |  | 0.102 | 0.018 | 2.36E-08 |
| rs17213127 | 3 | 45798226 | T | C | 0.019 | 0.003 | 1.99E-10 |  | 0.067 | 0.038 | 8.17E-02 |  | 0.301 | 0.049 | 8.80E-10 |
| rs17214952 | 3 | 46011436 | G | A | 0.024 | 0.002 | 1.58E-34 |  | 0.013 | 0.026 | 6.17E-01 |  | 0.336 | 0.027 | 1.57E-35 |
| rs17215008 | 3 | 46012279 | C | T | 0.024 | 0.002 | 3.69E-33 |  | 0.008 | 0.027 | 7.72E-01 |  | 0.335 | 0.027 | 2.34E-34 |
| rs17216717 | 3 | 46186323 | C | T | 0.017 | 0.002 | 3.59E-14 |  | 0.023 | 0.030 | 4.35E-01 |  | 0.238 | 0.031 | 3.30E-14 |
| rs17217831 | 3 | 46305441 | A | C | 0.017 | 0.002 | 1.20E-12 |  | 0.035 | 0.032 | 2.70E-01 |  | 0.239 | 0.034 | 2.02E-12 |
| rs17282391 | 3 | 46179481 | G | A | 0.017 | 0.002 | 4.86E-15 |  | 0.024 | 0.030 | 4.30E-01 |  | 0.247 | 0.031 | 4.30E-15 |
| rs17282797 | 3 | 46232765 | G | A | 0.017 | 0.002 | 2.60E-14 |  | 0.024 | 0.030 | 4.20E-01 |  | 0.239 | 0.031 | 2.48E-14 |
| rs17282922 | 3 | 46240172 | G | T | 0.017 | 0.002 | 3.46E-14 |  | 0.022 | 0.030 | 4.71E-01 |  | 0.239 | 0.031 | 2.87E-14 |
| rs17283712 | 3 | 46304064 | G | T | 0.017 | 0.002 | 9.58E-13 |  | 0.027 | 0.032 | 4.04E-01 |  | 0.243 | 0.034 | 1.04E-12 |
| rs17284138 | 3 | 46331852 | C | A | 0.017 | 0.002 | 2.07E-12 |  | 0.029 | 0.032 | 3.67E-01 |  | 0.240 | 0.034 | 2.55E-12 |
| rs17284187 | 3 | 46332999 | A | G | 0.018 | 0.003 | 6.04E-12 |  | 0.027 | 0.032 | 4.08E-01 |  | 0.239 | 0.035 | 5.83E-12 |
| rs17330872 | 3 | 46035097 | G | A | 0.024 | 0.002 | 6.34E-33 |  | 0.008 | 0.027 | 7.55E-01 |  | 0.335 | 0.027 | 4.38E-34 |
| rs17765307 | 3 | 46385161 | T | G | 0.016 | 0.002 | 4.26E-12 |  | 0.036 | 0.032 | 2.68E-01 |  | 0.235 | 0.034 | 7.32E-12 |
| rs187726344 | 3 | 46563790 | G | A | 0.023 | 0.004 | 2.35E-08 |  | 0.057 | 0.063 | 3.65E-01 |  | 0.355 | 0.064 | 3.41E-08 |
| rs1894387 | 3 | 46392089 | T | G | 0.016 | 0.002 | 3.43E-12 |  | 0.036 | 0.032 | 2.62E-01 |  | 0.237 | 0.034 | 6.01E-12 |
| rs1894388 | 3 | 46392131 | T | C | 0.016 | 0.002 | 5.30E-12 |  | 0.036 | 0.032 | 2.60E-01 |  | 0.234 | 0.034 | 9.43E-12 |
| rs1994492 | 3 | 45960646 | C | T | 0.024 | 0.002 | 3.65E-34 |  | 0.009 | 0.027 | 7.48E-01 |  | 0.345 | 0.028 | 2.27E-35 |
| rs1994493 | 3 | 45960700 | T | C | 0.024 | 0.002 | 4.50E-34 |  | 0.006 | 0.027 | 8.17E-01 |  | 0.345 | 0.028 | 2.23E-35 |
| rs2171531 | 3 | 45981171 | T | C | 0.024 | 0.002 | 7.37E-33 |  | 0.004 | 0.027 | 8.83E-01 |  | 0.336 | 0.028 | 3.28E-34 |
| rs2234358 | 3 | 45989044 | G | T | 0.008 | 0.001 | 1.84E-09 |  | 0.024 | 0.016 | 1.24E-01 |  | 0.106 | 0.018 | 5.91E-09 |
| rs2373087 | 3 | 45968043 | G | T | 0.024 | 0.002 | 9.00E-33 |  | 0.003 | 0.027 | 9.02E-01 |  | 0.336 | 0.028 | 3.77E-34 |
| rs28677778 | 3 | 46322171 | G | A | 0.016 | 0.002 | 6.72E-12 |  | 0.039 | 0.032 | 2.21E-01 |  | 0.224 | 0.033 | 1.39E-11 |
| rs3091314 | 3 | 46188753 | A | G | 0.017 | 0.002 | 2.97E-14 |  | 0.024 | 0.030 | 4.17E-01 |  | 0.239 | 0.031 | 2.87E-14 |
| rs3092957 | 3 | 46403961 | A | G | 0.016 | 0.002 | 6.33E-12 |  | 0.037 | 0.032 | 2.53E-01 |  | 0.233 | 0.034 | 1.16E-11 |
| rs3092958 | 3 | 46403681 | A | G | 0.016 | 0.002 | 7.95E-12 |  | 0.037 | 0.032 | 2.52E-01 |  | 0.232 | 0.034 | 1.46E-11 |
| rs3092959 | 3 | 46403468 | A | G | 0.016 | 0.002 | 6.13E-12 |  | 0.039 | 0.032 | 2.23E-01 |  | 0.233 | 0.034 | 1.26E-11 |
| rs3136672 | 3 | 46242785 | C | T | 0.016 | 0.002 | 1.97E-13 |  | 0.031 | 0.029 | 2.88E-01 |  | 0.216 | 0.030 | 3.01E-13 |
| rs3136673 | 3 | 46242616 | T | C | 0.016 | 0.002 | 6.02E-14 |  | 0.028 | 0.029 | 3.43E-01 |  | 0.228 | 0.030 | 7.44E-14 |
| rs3176824 | 3 | 46248385 | C | T | 0.016 | 0.002 | 1.92E-13 |  | 0.020 | 0.030 | 4.98E-01 |  | 0.233 | 0.032 | 1.56E-13 |
| rs3176825 | 3 | 46248224 | T | G | 0.016 | 0.002 | 1.69E-13 |  | 0.020 | 0.030 | 4.98E-01 |  | 0.234 | 0.032 | 1.36E-13 |
| rs3176826 | 3 | 46247584 | A | G | 0.017 | 0.002 | 1.64E-13 |  | 0.021 | 0.030 | 4.88E-01 |  | 0.234 | 0.032 | 1.36E-13 |
| rs3181076 | 3 | 46250733 | T | C | 0.018 | 0.003 | 1.72E-12 |  | 0.020 | 0.030 | 5.10E-01 |  | 0.227 | 0.032 | 1.14E-12 |
| rs3181078 | 3 | 46250584 | C | A | 0.016 | 0.002 | 2.03E-13 |  | 0.020 | 0.030 | 5.01E-01 |  | 0.233 | 0.032 | 1.63E-13 |
| rs33910087 | 3 | 46009487 | A | G | 0.024 | 0.002 | 2.16E-33 |  | 0.009 | 0.027 | 7.23E-01 |  | 0.336 | 0.027 | 1.59E-34 |
| rs33998492 | 3 | 46273598 | A | G | 0.018 | 0.002 | 3.55E-14 |  | 0.006 | 0.032 | 8.49E-01 |  | 0.259 | 0.034 | 1.18E-14 |
| rs34000569 | 3 | 45999209 | G | A | 0.024 | 0.002 | 7.89E-33 |  | 0.006 | 0.027 | 8.17E-01 |  | 0.334 | 0.027 | 4.42E-34 |
| rs34005848 | 3 | 46263745 | T | C | 0.019 | 0.002 | 3.24E-15 |  | 0.007 | 0.032 | 8.35E-01 |  | 0.264 | 0.033 | 1.01E-15 |
| rs34013035 | 3 | 46220682 | A | G | 0.016 | 0.002 | 1.96E-13 |  | 0.033 | 0.030 | 2.71E-01 |  | 0.222 | 0.030 | 3.18E-13 |
| rs34024447 | 3 | 46375737 | T | C | 0.016 | 0.002 | 4.20E-12 |  | 0.035 | 0.032 | 2.77E-01 |  | 0.236 | 0.034 | 6.99E-12 |
| rs34041956 | 3 | 46402734 | A | G | 0.016 | 0.002 | 6.86E-12 |  | 0.037 | 0.032 | 2.52E-01 |  | 0.233 | 0.034 | 1.26E-11 |
| rs34059564 | 3 | 46226165 | C | T | 0.016 | 0.002 | 7.02E-14 |  | 0.035 | 0.029 | 2.32E-01 |  | 0.220 | 0.030 | 1.31E-13 |
| rs34068335 | 3 | 45954339 | T | C | 0.024 | 0.002 | 4.67E-33 |  | 0.003 | 0.027 | 9.23E-01 |  | 0.345 | 0.028 | 1.78E-34 |
| rs34073838 | 3 | 46337692 | C | A | 0.017 | 0.002 | 2.22E-12 |  | 0.027 | 0.032 | 3.98E-01 |  | 0.240 | 0.034 | 2.50E-12 |
| rs34079287 | 3 | 46300870 | T | G | 0.017 | 0.002 | 9.42E-13 |  | 0.027 | 0.032 | 4.04E-01 |  | 0.243 | 0.034 | 1.02E-12 |
| rs34093271 | 3 | 46144981 | T | G | 0.018 | 0.002 | 3.36E-19 |  | 0.001 | 0.027 | 9.84E-01 |  | 0.282 | 0.031 | 4.72E-20 |
| rs34101673 | 3 | 46197286 | T | C | 0.017 | 0.002 | 4.92E-14 |  | 0.024 | 0.030 | 4.26E-01 |  | 0.238 | 0.032 | 4.68E-14 |
| rs34106161 | 3 | 46376169 | T | C | 0.016 | 0.002 | 3.96E-12 |  | 0.036 | 0.032 | 2.71E-01 |  | 0.236 | 0.034 | 6.73E-12 |
| rs34127208 | 3 | 46103680 | T | G | 0.023 | 0.002 | 2.63E-31 |  | 0.002 | 0.027 | 9.42E-01 |  | 0.328 | 0.028 | 1.13E-32 |
| rs34134191 | 3 | 46221531 | C | T | 0.017 | 0.002 | 3.01E-14 |  | 0.024 | 0.030 | 4.17E-01 |  | 0.239 | 0.031 | 2.90E-14 |
| rs34155121 | 3 | 46084742 | A | G | 0.020 | 0.002 | 8.39E-27 |  | 0.001 | 0.025 | 9.81E-01 |  | 0.277 | 0.025 | 5.20E-28 |
| rs34168660 | 3 | 46056162 | A | G | 0.023 | 0.002 | 6.68E-32 |  | 0.007 | 0.027 | 8.05E-01 |  | 0.324 | 0.027 | 4.29E-33 |
| rs34180919 | 3 | 46353199 | A | G | 0.016 | 0.002 | 4.29E-12 |  | 0.034 | 0.032 | 2.88E-01 |  | 0.236 | 0.034 | 6.88E-12 |
| rs34191675 | 3 | 46189448 | G | A | 0.018 | 0.002 | 5.44E-13 |  | 0.025 | 0.030 | 4.05E-01 |  | 0.227 | 0.031 | 4.75E-13 |
| rs34194160 | 3 | 46265854 | A | G | 0.019 | 0.002 | 4.57E-15 |  | 0.006 | 0.032 | 8.48E-01 |  | 0.265 | 0.033 | 1.40E-15 |
| rs34198655 | 3 | 46215107 | A | G | 0.016 | 0.002 | 2.03E-13 |  | 0.031 | 0.030 | 2.95E-01 |  | 0.223 | 0.031 | 3.03E-13 |
| rs34272024 | 3 | 46292339 | A | G | 0.016 | 0.002 | 2.65E-12 |  | 0.038 | 0.031 | 2.28E-01 |  | 0.224 | 0.032 | 5.28E-12 |
| rs34289272 | 3 | 46263244 | T | C | 0.019 | 0.002 | 3.76E-15 |  | 0.010 | 0.032 | 7.48E-01 |  | 0.263 | 0.033 | 1.44E-15 |
| rs34324101 | 3 | 46000728 | G | T | 0.024 | 0.002 | 1.34E-32 |  | 0.004 | 0.027 | 8.76E-01 |  | 0.334 | 0.027 | 6.29E-34 |
| rs34340501 | 3 | 46225216 | T | G | 0.017 | 0.002 | 3.04E-14 |  | 0.023 | 0.030 | 4.39E-01 |  | 0.239 | 0.031 | 2.75E-14 |
| rs34351442 | 3 | 46355492 | A | G | 0.016 | 0.002 | 4.46E-12 |  | 0.034 | 0.032 | 2.95E-01 |  | 0.236 | 0.034 | 7.00E-12 |
| rs34363105 | 3 | 46389745 | T | C | 0.016 | 0.002 | 4.07E-12 |  | 0.037 | 0.032 | 2.52E-01 |  | 0.235 | 0.034 | 7.41E-12 |
| rs34378541 | 3 | 46239649 | T | C | 0.017 | 0.002 | 3.24E-14 |  | 0.023 | 0.030 | 4.40E-01 |  | 0.239 | 0.031 | 2.93E-14 |
| rs34381952 | 3 | 45995748 | C | T | 0.024 | 0.002 | 7.56E-34 |  | 0.009 | 0.026 | 7.29E-01 |  | 0.334 | 0.027 | 5.23E-35 |
| rs34386754 | 3 | 46090013 | A | G | 0.014 | 0.002 | 1.69E-13 |  | 0.001 | 0.025 | 9.62E-01 |  | 0.221 | 0.029 | 4.67E-14 |
| rs34401473 | 3 | 46189772 | C | A | 0.016 | 0.002 | 8.24E-14 |  | 0.025 | 0.030 | 4.05E-01 |  | 0.233 | 0.031 | 8.44E-14 |
| rs34406035 | 3 | 46278919 | T | C | 0.017 | 0.002 | 4.07E-13 |  | 0.024 | 0.032 | 4.64E-01 |  | 0.246 | 0.034 | 3.66E-13 |
| rs34409248 | 3 | 46198071 | A | G | 0.017 | 0.002 | 5.24E-14 |  | 0.024 | 0.030 | 4.26E-01 |  | 0.238 | 0.032 | 4.99E-14 |
| rs34414382 | 3 | 46265190 | T | C | 0.019 | 0.002 | 3.32E-15 |  | 0.006 | 0.032 | 8.48E-01 |  | 0.264 | 0.033 | 1.00E-15 |
| rs34423195 | 3 | 46249722 | G | A | 0.016 | 0.002 | 1.70E-13 |  | 0.020 | 0.030 | 4.98E-01 |  | 0.234 | 0.032 | 1.37E-13 |
| rs34452002 | 3 | 46143187 | T | C | 0.018 | 0.002 | 3.93E-19 |  | 0.000 | 0.027 | 9.93E-01 |  | 0.282 | 0.031 | 5.43E-20 |
| rs34460587 | 3 | 46142464 | T | C | 0.023 | 0.002 | 4.97E-31 |  | 0.000 | 0.027 | 9.87E-01 |  | 0.327 | 0.028 | 1.90E-32 |
| rs34473395 | 3 | 46392976 | T | C | 0.016 | 0.002 | 6.33E-12 |  | 0.036 | 0.032 | 2.61E-01 |  | 0.233 | 0.034 | 1.12E-11 |
| rs34493660 | 3 | 46052800 | A | G | 0.023 | 0.002 | 9.51E-32 |  | 0.010 | 0.027 | 7.19E-01 |  | 0.322 | 0.027 | 8.32E-33 |
| rs34523728 | 3 | 46219833 | A | C | 0.017 | 0.002 | 3.47E-14 |  | 0.024 | 0.030 | 4.17E-01 |  | 0.238 | 0.031 | 3.35E-14 |
| rs34531115 | 3 | 46257696 | C | T | 0.016 | 0.002 | 1.74E-13 |  | 0.019 | 0.030 | 5.37E-01 |  | 0.234 | 0.032 | 1.27E-13 |
| rs34558763 | 3 | 46189924 | T | C | 0.017 | 0.002 | 2.67E-14 |  | 0.024 | 0.030 | 4.17E-01 |  | 0.239 | 0.031 | 2.57E-14 |
| rs34567015 | 3 | 46202212 | A | G | 0.017 | 0.002 | 4.01E-14 |  | 0.025 | 0.030 | 4.11E-01 |  | 0.238 | 0.031 | 3.96E-14 |
| rs34570200 | 3 | 46220500 | T | C | 0.017 | 0.002 | 3.53E-14 |  | 0.024 | 0.030 | 4.17E-01 |  | 0.238 | 0.031 | 3.42E-14 |
| rs34677490 | 3 | 46270748 | T | G | 0.019 | 0.002 | 3.22E-15 |  | 0.006 | 0.032 | 8.52E-01 |  | 0.264 | 0.033 | 9.62E-16 |
| rs34679077 | 3 | 46087992 | A | G | 0.023 | 0.002 | 1.13E-31 |  | 0.000 | 0.027 | 9.95E-01 |  | 0.330 | 0.028 | 3.90E-33 |
| rs34692251 | 3 | 46273727 | A | G | 0.018 | 0.002 | 7.88E-14 |  | 0.008 | 0.032 | 7.91E-01 |  | 0.255 | 0.034 | 3.08E-14 |
| rs34693386 | 3 | 46195402 | T | C | 0.017 | 0.002 | 2.94E-14 |  | 0.024 | 0.030 | 4.35E-01 |  | 0.240 | 0.032 | 2.69E-14 |
| rs34709208 | 3 | 46374653 | A | G | 0.017 | 0.002 | 2.32E-12 |  | 0.035 | 0.032 | 2.83E-01 |  | 0.239 | 0.034 | 3.76E-12 |
| rs34745455 | 3 | 46267333 | C | T | 0.019 | 0.002 | 3.49E-15 |  | 0.006 | 0.032 | 8.52E-01 |  | 0.264 | 0.033 | 1.05E-15 |
| rs34754340 | 3 | 46041837 | T | C | 0.024 | 0.002 | 9.67E-33 |  | 0.010 | 0.027 | 7.17E-01 |  | 0.333 | 0.027 | 7.77E-34 |
| rs34759782 | 3 | 46260444 | A | G | 0.016 | 0.002 | 5.42E-13 |  | 0.025 | 0.029 | 3.92E-01 |  | 0.213 | 0.030 | 6.03E-13 |
| rs34766614 | 3 | 46107601 | G | A | 0.023 | 0.002 | 3.12E-31 |  | 0.001 | 0.027 | 9.68E-01 |  | 0.328 | 0.028 | 1.24E-32 |
| rs34836513 | 3 | 46046459 | A | G | 0.024 | 0.002 | 7.87E-33 |  | 0.010 | 0.027 | 7.01E-01 |  | 0.334 | 0.027 | 6.64E-34 |
| rs34849862 | 3 | 46001367 | A | C | 0.024 | 0.002 | 1.29E-32 |  | 0.004 | 0.027 | 8.70E-01 |  | 0.334 | 0.027 | 6.13E-34 |
| rs34865316 | 3 | 46247456 | G | A | 0.016 | 0.002 | 1.70E-13 |  | 0.020 | 0.030 | 4.97E-01 |  | 0.234 | 0.032 | 1.37E-13 |
| rs34867672 | 3 | 46088282 | C | T | 0.020 | 0.002 | 8.09E-27 |  | 0.001 | 0.025 | 9.62E-01 |  | 0.277 | 0.025 | 5.34E-28 |
| rs34870159 | 3 | 46189490 | C | T | 0.017 | 0.002 | 2.47E-14 |  | 0.024 | 0.030 | 4.22E-01 |  | 0.237 | 0.031 | 2.34E-14 |
| rs34897745 | 3 | 46198376 | C | A | 0.017 | 0.002 | 5.50E-14 |  | 0.021 | 0.030 | 4.77E-01 |  | 0.238 | 0.032 | 4.54E-14 |
| rs34919616 | 3 | 46250008 | A | G | 0.017 | 0.002 | 1.45E-13 |  | 0.023 | 0.030 | 4.39E-01 |  | 0.234 | 0.032 | 1.36E-13 |
| rs34920132 | 3 | 46226769 | G | A | 0.016 | 0.002 | 8.16E-14 |  | 0.034 | 0.029 | 2.47E-01 |  | 0.219 | 0.030 | 1.44E-13 |
| rs34924300 | 3 | 46088336 | T | G | 0.020 | 0.002 | 9.80E-27 |  | 0.001 | 0.025 | 9.62E-01 |  | 0.276 | 0.025 | 6.53E-28 |
| rs34985947 | 3 | 46260008 | T | C | 0.016 | 0.002 | 6.00E-13 |  | 0.023 | 0.029 | 4.33E-01 |  | 0.215 | 0.030 | 5.95E-13 |
| rs34988015 | 3 | 46356048 | C | T | 0.018 | 0.003 | 1.28E-11 |  | 0.034 | 0.032 | 2.97E-01 |  | 0.233 | 0.035 | 1.79E-11 |
| rs35035328 | 3 | 46276078 | C | T | 0.017 | 0.002 | 6.50E-13 |  | 0.019 | 0.032 | 5.61E-01 |  | 0.244 | 0.034 | 4.65E-13 |
| rs35110864 | 3 | 46154457 | A | G | 0.017 | 0.002 | 1.79E-18 |  | 0.001 | 0.027 | 9.77E-01 |  | 0.280 | 0.031 | 2.78E-19 |
| rs35117954 | 3 | 46338831 | G | A | 0.017 | 0.002 | 2.04E-12 |  | 0.029 | 0.032 | 3.76E-01 |  | 0.240 | 0.034 | 2.44E-12 |
| rs35161099 | 3 | 46067507 | G | T | 0.023 | 0.002 | 2.38E-31 |  | 0.001 | 0.027 | 9.85E-01 |  | 0.328 | 0.028 | 8.83E-33 |
| rs35162796 | 3 | 46204379 | T | C | 0.017 | 0.002 | 5.44E-14 |  | 0.024 | 0.030 | 4.17E-01 |  | 0.237 | 0.032 | 5.33E-14 |
| rs35195804 | 3 | 46260195 | G | A | 0.016 | 0.002 | 8.24E-13 |  | 0.022 | 0.029 | 4.57E-01 |  | 0.214 | 0.030 | 7.70E-13 |
| rs35203745 | 3 | 46322289 | C | T | 0.016 | 0.002 | 5.12E-12 |  | 0.040 | 0.032 | 2.07E-01 |  | 0.224 | 0.033 | 1.12E-11 |
| rs35209528 | 3 | 46003496 | C | T | 0.024 | 0.002 | 1.32E-32 |  | 0.004 | 0.027 | 8.70E-01 |  | 0.333 | 0.027 | 6.32E-34 |
| rs35218998 | 3 | 46328715 | C | T | 0.017 | 0.002 | 1.49E-12 |  | 0.029 | 0.032 | 3.67E-01 |  | 0.241 | 0.034 | 1.82E-12 |
| rs35280891 | 3 | 45951647 | A | G | 0.017 | 0.002 | 2.15E-18 |  | 0.005 | 0.027 | 8.55E-01 |  | 0.280 | 0.031 | 4.56E-19 |
| rs35354367 | 3 | 46250799 | G | A | 0.016 | 0.002 | 1.88E-13 |  | 0.020 | 0.030 | 5.11E-01 |  | 0.234 | 0.032 | 1.46E-13 |
| rs35373513 | 3 | 46312953 | T | C | 0.017 | 0.002 | 1.12E-12 |  | 0.029 | 0.032 | 3.63E-01 |  | 0.242 | 0.034 | 1.39E-12 |
| rs35413141 | 3 | 46389649 | A | G | 0.016 | 0.002 | 4.05E-12 |  | 0.037 | 0.032 | 2.54E-01 |  | 0.235 | 0.034 | 7.31E-12 |
| rs35429781 | 3 | 46253607 | C | A | 0.016 | 0.002 | 1.79E-13 |  | 0.020 | 0.030 | 5.10E-01 |  | 0.234 | 0.032 | 1.40E-13 |
| rs35434266 | 3 | 46206999 | T | G | 0.017 | 0.002 | 5.20E-14 |  | 0.024 | 0.030 | 4.32E-01 |  | 0.238 | 0.032 | 4.87E-14 |
| rs35477280 | 3 | 45974092 | A | G | 0.024 | 0.002 | 5.83E-33 |  | 0.006 | 0.027 | 8.18E-01 |  | 0.337 | 0.028 | 3.22E-34 |
| rs35481399 | 3 | 46265580 | A | C | 0.017 | 0.002 | 3.98E-14 |  | 0.011 | 0.031 | 7.09E-01 |  | 0.239 | 0.031 | 1.83E-14 |
| rs35501575 | 3 | 45993645 | T | C | 0.024 | 0.002 | 1.03E-32 |  | 0.005 | 0.027 | 8.40E-01 |  | 0.334 | 0.027 | 5.40E-34 |
| rs35511592 | 3 | 46334846 | A | G | 0.017 | 0.002 | 1.95E-12 |  | 0.029 | 0.032 | 3.76E-01 |  | 0.240 | 0.034 | 2.33E-12 |
| rs35513549 | 3 | 46408180 | A | C | 0.016 | 0.002 | 8.53E-12 |  | 0.038 | 0.032 | 2.43E-01 |  | 0.232 | 0.034 | 1.62E-11 |
| rs35516580 | 3 | 46107498 | G | A | 0.023 | 0.002 | 8.41E-32 |  | 0.001 | 0.027 | 9.71E-01 |  | 0.331 | 0.028 | 3.12E-33 |
| rs35525815 | 3 | 46006269 | T | C | 0.024 | 0.002 | 2.59E-34 |  | 0.011 | 0.027 | 6.72E-01 |  | 0.336 | 0.027 | 2.12E-35 |
| rs35539222 | 3 | 46154272 | C | T | 0.017 | 0.002 | 1.80E-18 |  | 0.001 | 0.027 | 9.77E-01 |  | 0.280 | 0.031 | 2.79E-19 |
| rs35560301 | 3 | 46220506 | G | T | 0.017 | 0.002 | 3.55E-14 |  | 0.024 | 0.030 | 4.17E-01 |  | 0.238 | 0.031 | 3.44E-14 |
| rs35566550 | 3 | 46210512 | T | G | 0.017 | 0.002 | 4.46E-14 |  | 0.023 | 0.030 | 4.35E-01 |  | 0.239 | 0.032 | 4.12E-14 |
| rs35587265 | 3 | 46259296 | G | A | 0.018 | 0.003 | 1.58E-12 |  | 0.019 | 0.030 | 5.29E-01 |  | 0.228 | 0.032 | 9.82E-13 |
| rs35601704 | 3 | 46389547 | A | G | 0.016 | 0.002 | 4.23E-12 |  | 0.036 | 0.032 | 2.63E-01 |  | 0.235 | 0.034 | 7.38E-12 |
| rs35613615 | 3 | 46265243 | C | T | 0.019 | 0.002 | 3.32E-15 |  | 0.006 | 0.032 | 8.48E-01 |  | 0.264 | 0.033 | 1.00E-15 |
| rs35614049 | 3 | 46255476 | C | T | 0.016 | 0.002 | 1.71E-13 |  | 0.017 | 0.030 | 5.65E-01 |  | 0.235 | 0.032 | 1.16E-13 |
| rs35617677 | 3 | 46258740 | T | C | 0.016 | 0.002 | 1.75E-13 |  | 0.019 | 0.030 | 5.35E-01 |  | 0.234 | 0.032 | 1.29E-13 |
| rs35646896 | 3 | 46320120 | T | G | 0.017 | 0.002 | 1.66E-12 |  | 0.029 | 0.032 | 3.67E-01 |  | 0.241 | 0.034 | 2.03E-12 |
| rs35669129 | 3 | 46055250 | A | G | 0.023 | 0.002 | 2.35E-31 |  | 0.010 | 0.027 | 7.20E-01 |  | 0.320 | 0.027 | 2.14E-32 |
| rs35678191 | 3 | 46271399 | C | T | 0.017 | 0.002 | 6.86E-14 |  | 0.011 | 0.031 | 7.13E-01 |  | 0.237 | 0.031 | 3.18E-14 |
| rs35685805 | 3 | 46202778 | C | T | 0.017 | 0.002 | 4.11E-14 |  | 0.025 | 0.030 | 4.12E-01 |  | 0.238 | 0.031 | 4.06E-14 |
| rs35751180 | 3 | 46056173 | T | C | 0.024 | 0.002 | 1.10E-32 |  | 0.007 | 0.027 | 7.87E-01 |  | 0.334 | 0.028 | 6.92E-34 |
| rs35754688 | 3 | 46187706 | T | C | 0.017 | 0.002 | 8.19E-14 |  | 0.027 | 0.030 | 3.72E-01 |  | 0.233 | 0.031 | 9.28E-14 |
| rs35772789 | 3 | 46095104 | G | A | 0.023 | 0.002 | 1.72E-31 |  | 0.000 | 0.027 | 9.98E-01 |  | 0.329 | 0.028 | 6.01E-33 |
| rs35775079 | 3 | 46262111 | T | C | 0.017 | 0.002 | 1.49E-13 |  | 0.019 | 0.030 | 5.32E-01 |  | 0.235 | 0.032 | 1.10E-13 |
| rs35827997 | 3 | 46001720 | G | T | 0.024 | 0.002 | 4.48E-34 |  | 0.010 | 0.026 | 7.02E-01 |  | 0.335 | 0.027 | 3.35E-35 |
| rs35831747 | 3 | 45970391 | A | G | 0.024 | 0.002 | 9.76E-33 |  | 0.005 | 0.027 | 8.63E-01 |  | 0.336 | 0.028 | 4.71E-34 |
| rs35855315 | 3 | 46006239 | G | A | 0.024 | 0.002 | 2.66E-34 |  | 0.011 | 0.027 | 6.72E-01 |  | 0.335 | 0.027 | 2.18E-35 |
| rs35883205 | 3 | 46053968 | G | A | 0.024 | 0.002 | 9.12E-33 |  | 0.009 | 0.027 | 7.29E-01 |  | 0.334 | 0.028 | 7.00E-34 |
| rs35919278 | 3 | 46213594 | G | A | 0.017 | 0.002 | 4.30E-14 |  | 0.024 | 0.030 | 4.32E-01 |  | 0.239 | 0.032 | 4.01E-14 |
| rs35942803 | 3 | 46208783 | C | T | 0.017 | 0.002 | 5.21E-14 |  | 0.024 | 0.030 | 4.32E-01 |  | 0.238 | 0.032 | 4.88E-14 |
| rs35943069 | 3 | 46391648 | G | T | 0.016 | 0.002 | 3.91E-12 |  | 0.037 | 0.032 | 2.51E-01 |  | 0.236 | 0.034 | 7.14E-12 |
| rs35984988 | 3 | 46261256 | G | A | 0.016 | 0.002 | 6.08E-13 |  | 0.023 | 0.029 | 4.34E-01 |  | 0.215 | 0.030 | 6.02E-13 |
| rs36000011 | 3 | 46265535 | A | C | 0.018 | 0.002 | 2.67E-14 |  | 0.015 | 0.031 | 6.32E-01 |  | 0.238 | 0.031 | 1.45E-14 |
| rs36010446 | 3 | 46226725 | A | G | 0.017 | 0.002 | 3.68E-14 |  | 0.023 | 0.030 | 4.50E-01 |  | 0.238 | 0.031 | 3.25E-14 |
| rs36039366 | 3 | 46003537 | A | G | 0.024 | 0.002 | 3.96E-34 |  | 0.010 | 0.026 | 7.19E-01 |  | 0.335 | 0.027 | 2.75E-35 |
| rs36078103 | 3 | 46311782 | T | C | 0.017 | 0.002 | 1.18E-12 |  | 0.029 | 0.032 | 3.63E-01 |  | 0.242 | 0.034 | 1.46E-12 |
| rs36122610 | 3 | 46022833 | A | G | 0.024 | 0.002 | 5.37E-33 |  | 0.008 | 0.027 | 7.52E-01 |  | 0.334 | 0.027 | 3.71E-34 |
| rs36127773 | 3 | 46216245 | G | A | 0.013 | 0.002 | 9.87E-09 |  | 0.024 | 0.030 | 4.17E-01 |  | 0.208 | 0.037 | 1.25E-08 |
| rs36189995 | 3 | 46190064 | G | A | 0.012 | 0.002 | 1.75E-08 |  | 0.025 | 0.030 | 4.07E-01 |  | 0.202 | 0.036 | 2.29E-08 |
| rs371395769 | 3 | 46190374 | C | T | 0.013 | 0.002 | 4.14E-08 |  | 0.024 | 0.030 | 4.17E-01 |  | 0.202 | 0.037 | 5.06E-08 |
| rs3749461 | 3 | 46395313 | G | A | 0.016 | 0.002 | 6.42E-12 |  | 0.036 | 0.032 | 2.59E-01 |  | 0.233 | 0.034 | 1.14E-11 |
| rs3851347 | 3 | 46053778 | G | A | 0.008 | 0.001 | 6.01E-10 |  | 0.000 | 0.017 | 9.99E-01 |  | 0.121 | 0.019 | 2.31E-10 |
| rs3918354 | 3 | 46393463 | G | A | 0.017 | 0.003 | 1.51E-11 |  | 0.036 | 0.032 | 2.61E-01 |  | 0.231 | 0.035 | 2.55E-11 |
| rs3918365 | 3 | 46398364 | G | A | 0.016 | 0.002 | 6.33E-12 |  | 0.037 | 0.032 | 2.54E-01 |  | 0.233 | 0.034 | 1.15E-11 |
| rs3918368 | 3 | 46403240 | A | G | 0.016 | 0.002 | 7.00E-12 |  | 0.037 | 0.032 | 2.52E-01 |  | 0.233 | 0.034 | 1.28E-11 |
| rs41289616 | 3 | 45997263 | C | T | 0.024 | 0.002 | 6.83E-34 |  | 0.010 | 0.027 | 6.96E-01 |  | 0.334 | 0.027 | 5.33E-35 |
| rs41289622 | 3 | 46014545 | G | T | 0.024 | 0.002 | 2.93E-33 |  | 0.008 | 0.027 | 7.71E-01 |  | 0.336 | 0.027 | 1.84E-34 |
| rs41432345 | 3 | 46250374 | C | T | 0.016 | 0.002 | 1.63E-13 |  | 0.028 | 0.029 | 3.42E-01 |  | 0.220 | 0.030 | 2.07E-13 |
| rs41537946 | 3 | 46260860 | T | C | 0.017 | 0.002 | 7.61E-12 |  | 0.023 | 0.029 | 4.34E-01 |  | 0.207 | 0.030 | 6.64E-12 |
| rs4234453 | 3 | 46084949 | A | C | 0.022 | 0.002 | 3.17E-26 |  | 0.001 | 0.025 | 9.81E-01 |  | 0.278 | 0.025 | 1.02E-27 |
| rs4388012 | 3 | 45996501 | G | A | 0.024 | 0.002 | 4.98E-34 |  | 0.009 | 0.026 | 7.27E-01 |  | 0.335 | 0.027 | 3.42E-35 |
| rs4443214 | 3 | 46177864 | C | T | 0.018 | 0.002 | 7.24E-20 |  | 0.013 | 0.027 | 6.26E-01 |  | 0.257 | 0.028 | 2.53E-20 |
| rs4473594 | 3 | 46337356 | A | G | 0.017 | 0.002 | 2.03E-12 |  | 0.029 | 0.032 | 3.76E-01 |  | 0.240 | 0.034 | 2.44E-12 |
| rs4493469 | 3 | 46177992 | C | T | 0.020 | 0.002 | 4.21E-19 |  | 0.003 | 0.028 | 9.16E-01 |  | 0.270 | 0.029 | 4.90E-20 |
| rs4682799 | 3 | 45993606 | C | T | 0.007 | 0.001 | 6.71E-09 |  | 0.024 | 0.016 | 1.35E-01 |  | 0.103 | 0.018 | 2.03E-08 |
| rs4682800 | 3 | 46002005 | C | A | 0.007 | 0.001 | 6.84E-09 |  | 0.024 | 0.016 | 1.30E-01 |  | 0.103 | 0.018 | 2.13E-08 |
| rs4683153 | 3 | 45974968 | T | G | 0.007 | 0.001 | 4.64E-08 |  | 0.022 | 0.016 | 1.72E-01 |  | 0.098 | 0.018 | 1.18E-07 |
| rs4683163 | 3 | 46084372 | C | A | 0.020 | 0.002 | 9.91E-27 |  | 0.000 | 0.025 | 9.94E-01 |  | 0.276 | 0.025 | 5.98E-28 |
| rs4987053 | 3 | 46306700 | C | T | 0.017 | 0.002 | 1.42E-12 |  | 0.044 | 0.032 | 1.69E-01 |  | 0.230 | 0.033 | 3.67E-12 |
| rs55875328 | 3 | 46051702 | A | G | 0.023 | 0.002 | 6.42E-32 |  | 0.012 | 0.027 | 6.45E-01 |  | 0.322 | 0.027 | 7.24E-33 |
| rs56332428 | 3 | 45989873 | T | C | 0.024 | 0.002 | 1.86E-33 |  | 0.009 | 0.026 | 7.29E-01 |  | 0.335 | 0.027 | 1.33E-34 |
| rs57115330 | 3 | 46255262 | C | T | 0.016 | 0.002 | 8.23E-13 |  | 0.023 | 0.029 | 4.40E-01 |  | 0.214 | 0.030 | 8.06E-13 |
| rs61650989 | 3 | 46054156 | T | G | 0.023 | 0.002 | 1.00E-31 |  | 0.009 | 0.027 | 7.22E-01 |  | 0.322 | 0.027 | 8.70E-33 |
| rs67200151 | 3 | 46051774 | A | G | 0.023 | 0.002 | 9.33E-32 |  | 0.009 | 0.027 | 7.26E-01 |  | 0.322 | 0.027 | 8.00E-33 |
| rs6764042 | 3 | 46059139 | G | T | 0.008 | 0.001 | 5.49E-10 |  | 0.000 | 0.017 | 9.87E-01 |  | 0.122 | 0.019 | 2.14E-10 |
| rs6765904 | 3 | 46195881 | G | A | 0.016 | 0.002 | 1.64E-13 |  | 0.029 | 0.030 | 3.25E-01 |  | 0.227 | 0.031 | 2.20E-13 |
| rs67676925 | 3 | 46274259 | C | T | 0.017 | 0.002 | 9.39E-14 |  | 0.014 | 0.031 | 6.46E-01 |  | 0.252 | 0.033 | 5.14E-14 |
| rs6781121 | 3 | 45975093 | C | A | 0.007 | 0.001 | 4.54E-08 |  | 0.022 | 0.016 | 1.70E-01 |  | 0.098 | 0.018 | 1.17E-07 |
| rs6793370 | 3 | 46312847 | G | A | 0.016 | 0.002 | 1.93E-12 |  | 0.040 | 0.032 | 2.05E-01 |  | 0.229 | 0.033 | 4.21E-12 |
| rs67937868 | 3 | 46049827 | T | G | 0.023 | 0.002 | 8.40E-32 |  | 0.011 | 0.027 | 6.93E-01 |  | 0.323 | 0.027 | 8.04E-33 |
| rs6803341 | 3 | 46315657 | G | A | 0.016 | 0.002 | 2.36E-12 |  | 0.041 | 0.032 | 1.95E-01 |  | 0.228 | 0.033 | 5.42E-12 |
| rs71325095 | 3 | 45989921 | T | G | 0.024 | 0.002 | 1.25E-33 |  | 0.011 | 0.027 | 6.70E-01 |  | 0.336 | 0.027 | 1.10E-34 |
| rs71325100 | 3 | 46013832 | T | C | 0.024 | 0.002 | 3.20E-33 |  | 0.008 | 0.027 | 7.53E-01 |  | 0.335 | 0.027 | 2.16E-34 |
| rs71325101 | 3 | 46015569 | C | T | 0.025 | 0.002 | 1.02E-34 |  | 0.011 | 0.027 | 6.82E-01 |  | 0.338 | 0.027 | 7.73E-36 |
| rs71325102 | 3 | 46015933 | C | T | 0.024 | 0.002 | 2.27E-34 |  | 0.013 | 0.026 | 6.29E-01 |  | 0.335 | 0.027 | 2.18E-35 |
| rs71327003 | 3 | 46036521 | T | C | 0.017 | 0.002 | 2.76E-17 |  | 0.009 | 0.027 | 7.47E-01 |  | 0.277 | 0.032 | 8.55E-18 |
| rs71327006 | 3 | 46058908 | A | G | 0.023 | 0.002 | 2.52E-31 |  | 0.000 | 0.027 | 9.98E-01 |  | 0.328 | 0.028 | 9.01E-33 |
| rs71327014 | 3 | 46101466 | T | C | 0.023 | 0.002 | 1.67E-31 |  | 0.001 | 0.027 | 9.76E-01 |  | 0.329 | 0.028 | 6.28E-33 |
| rs71327024 | 3 | 46140073 | T | G | 0.023 | 0.002 | 1.63E-30 |  | 0.000 | 0.027 | 9.98E-01 |  | 0.324 | 0.028 | 6.34E-32 |
| rs71327025 | 3 | 46147504 | G | A | 0.018 | 0.002 | 3.05E-19 |  | 0.000 | 0.027 | 9.96E-01 |  | 0.283 | 0.031 | 4.14E-20 |
| rs71327035 | 3 | 46194528 | C | T | 0.017 | 0.002 | 1.19E-12 |  | 0.032 | 0.029 | 2.76E-01 |  | 0.216 | 0.031 | 1.71E-12 |
| rs71327036 | 3 | 46194589 | A | C | 0.016 | 0.002 | 1.29E-13 |  | 0.032 | 0.030 | 2.89E-01 |  | 0.223 | 0.030 | 1.95E-13 |
| rs71327038 | 3 | 46225995 | C | A | 0.017 | 0.002 | 3.90E-14 |  | 0.023 | 0.030 | 4.40E-01 |  | 0.238 | 0.031 | 3.53E-14 |
| rs71327039 | 3 | 46257296 | C | A | 0.016 | 0.002 | 1.71E-13 |  | 0.019 | 0.030 | 5.37E-01 |  | 0.234 | 0.032 | 1.25E-13 |
| rs71327040 | 3 | 46263208 | T | G | 0.019 | 0.002 | 3.69E-15 |  | 0.006 | 0.032 | 8.51E-01 |  | 0.264 | 0.033 | 1.11E-15 |
| rs71327041 | 3 | 46267582 | T | C | 0.019 | 0.002 | 2.68E-15 |  | 0.008 | 0.032 | 8.01E-01 |  | 0.264 | 0.033 | 8.95E-16 |
| rs71327042 | 3 | 46268013 | C | T | 0.019 | 0.002 | 3.02E-15 |  | 0.006 | 0.032 | 8.51E-01 |  | 0.268 | 0.033 | 9.00E-16 |
| rs71327047 | 3 | 46310840 | C | T | 0.017 | 0.002 | 6.22E-13 |  | 0.029 | 0.032 | 3.66E-01 |  | 0.248 | 0.035 | 7.50E-13 |
| rs71327048 | 3 | 46350056 | C | A | 0.017 | 0.002 | 2.44E-12 |  | 0.033 | 0.032 | 3.10E-01 |  | 0.239 | 0.034 | 3.60E-12 |
| rs71327052 | 3 | 46378309 | G | A | 0.016 | 0.002 | 4.27E-12 |  | 0.035 | 0.032 | 2.72E-01 |  | 0.235 | 0.034 | 7.24E-12 |
| rs71327053 | 3 | 46381507 | T | C | 0.016 | 0.002 | 4.15E-12 |  | 0.036 | 0.032 | 2.69E-01 |  | 0.236 | 0.034 | 7.10E-12 |
| rs71327054 | 3 | 46381772 | T | C | 0.016 | 0.002 | 4.33E-12 |  | 0.036 | 0.032 | 2.69E-01 |  | 0.235 | 0.034 | 7.42E-12 |
| rs71327057 | 3 | 46402645 | C | A | 0.016 | 0.002 | 6.89E-12 |  | 0.037 | 0.032 | 2.59E-01 |  | 0.233 | 0.034 | 1.23E-11 |
| rs71615438 | 3 | 46039868 | G | A | 0.024 | 0.002 | 1.34E-32 |  | 0.009 | 0.027 | 7.25E-01 |  | 0.332 | 0.027 | 1.07E-33 |
| rs71615445 | 3 | 46377005 | T | C | 0.016 | 0.002 | 4.19E-12 |  | 0.035 | 0.032 | 2.73E-01 |  | 0.236 | 0.034 | 7.09E-12 |
| rs72901034 | 3 | 46085321 | G | A | 0.022 | 0.002 | 1.27E-25 |  | 0.001 | 0.025 | 9.73E-01 |  | 0.274 | 0.025 | 3.99E-27 |
| rs7427952 | 3 | 46386132 | G | A | 0.016 | 0.002 | 4.23E-12 |  | 0.036 | 0.032 | 2.60E-01 |  | 0.235 | 0.034 | 7.48E-12 |
| rs74586549 | 3 | 45926043 | T | C | 0.017 | 0.002 | 1.38E-18 |  | 0.005 | 0.026 | 8.36E-01 |  | 0.271 | 0.030 | 3.02E-19 |
| rs75928798 | 3 | 45962603 | G | A | 0.024 | 0.002 | 1.00E-33 |  | 0.007 | 0.027 | 8.02E-01 |  | 0.343 | 0.028 | 5.38E-35 |
| rs7620586 | 3 | 46267737 | G | T | 0.017 | 0.002 | 4.21E-14 |  | 0.012 | 0.031 | 6.97E-01 |  | 0.238 | 0.031 | 1.99E-14 |
| rs7623190 | 3 | 46068325 | C | T | 0.008 | 0.001 | 6.45E-10 |  | 0.000 | 0.017 | 1.00E+00 |  | 0.122 | 0.019 | 2.47E-10 |
| rs7624155 | 3 | 46269136 | C | T | 0.017 | 0.002 | 3.51E-14 |  | 0.013 | 0.031 | 6.82E-01 |  | 0.237 | 0.031 | 1.71E-14 |
| rs76281521 | 3 | 46049764 | A | G | 0.024 | 0.002 | 2.69E-33 |  | 0.013 | 0.027 | 6.32E-01 |  | 0.337 | 0.028 | 2.81E-34 |
| rs7628489 | 3 | 46267875 | A | G | 0.013 | 0.002 | 2.81E-08 |  | 0.013 | 0.031 | 6.80E-01 |  | 0.202 | 0.036 | 2.18E-08 |
| rs7631551 | 3 | 46186310 | A | C | 0.016 | 0.002 | 1.54E-13 |  | 0.030 | 0.030 | 3.09E-01 |  | 0.223 | 0.030 | 2.17E-13 |
| rs7631853 | 3 | 46306474 | C | T | 0.017 | 0.002 | 8.55E-13 |  | 0.044 | 0.032 | 1.66E-01 |  | 0.232 | 0.033 | 2.23E-12 |
| rs7642229 | 3 | 46090557 | G | A | 0.020 | 0.002 | 1.23E-26 |  | 0.001 | 0.025 | 9.58E-01 |  | 0.276 | 0.025 | 8.35E-28 |
| rs7642320 | 3 | 46090622 | G | A | 0.020 | 0.002 | 1.02E-26 |  | 0.001 | 0.025 | 9.68E-01 |  | 0.277 | 0.025 | 6.66E-28 |
| rs7649905 | 3 | 46316005 | C | T | 0.016 | 0.002 | 2.44E-12 |  | 0.041 | 0.032 | 1.95E-01 |  | 0.228 | 0.033 | 5.60E-12 |
| rs7651539 | 3 | 46268079 | T | C | 0.017 | 0.002 | 4.21E-14 |  | 0.012 | 0.031 | 6.97E-01 |  | 0.238 | 0.031 | 1.99E-14 |
| rs7652478 | 3 | 46090477 | A | G | 0.020 | 0.002 | 1.26E-26 |  | 0.002 | 0.025 | 9.51E-01 |  | 0.276 | 0.025 | 8.72E-28 |
| rs7653372 | 3 | 45927759 | C | T | 0.016 | 0.002 | 1.04E-17 |  | 0.009 | 0.026 | 7.38E-01 |  | 0.262 | 0.030 | 3.18E-18 |
| rs76597151 | 3 | 46001227 | A | G | 0.024 | 0.002 | 4.65E-34 |  | 0.010 | 0.026 | 7.18E-01 |  | 0.335 | 0.027 | 3.29E-35 |
| rs76632867 | 3 | 46151558 | G | A | 0.025 | 0.004 | 1.74E-12 |  | 0.035 | 0.053 | 5.15E-01 |  | 0.453 | 0.064 | 1.42E-12 |
| rs76836071 | 3 | 46344999 | T | C | 0.017 | 0.002 | 2.10E-12 |  | 0.032 | 0.032 | 3.25E-01 |  | 0.239 | 0.034 | 2.94E-12 |
| rs77902290 | 3 | 45976662 | T | C | 0.024 | 0.002 | 4.56E-33 |  | 0.004 | 0.027 | 8.77E-01 |  | 0.337 | 0.028 | 2.02E-34 |
| rs78124692 | 3 | 46202237 | T | C | 0.017 | 0.002 | 4.01E-14 |  | 0.025 | 0.030 | 4.12E-01 |  | 0.238 | 0.031 | 3.96E-14 |
| rs78919248 | 3 | 46279150 | T | C | 0.024 | 0.003 | 1.04E-17 |  | 0.026 | 0.038 | 4.97E-01 |  | 0.348 | 0.040 | 6.28E-18 |
| rs79457566 | 3 | 45674359 | G | A | 0.016 | 0.003 | 2.01E-08 |  | 0.075 | 0.037 | 4.21E-02 |  | 0.256 | 0.048 | 1.19E-07 |
| rs79509816 | 3 | 46375657 | A | G | 0.016 | 0.002 | 3.39E-12 |  | 0.035 | 0.032 | 2.80E-01 |  | 0.237 | 0.034 | 5.56E-12 |
| rs79948053 | 3 | 46211060 | A | G | 0.017 | 0.002 | 3.67E-14 |  | 0.024 | 0.030 | 4.28E-01 |  | 0.238 | 0.031 | 3.44E-14 |
| rs80007414 | 3 | 46202238 | G | A | 0.017 | 0.002 | 4.01E-14 |  | 0.025 | 0.030 | 4.12E-01 |  | 0.238 | 0.031 | 3.96E-14 |
| rs9311380 | 3 | 45996047 | C | T | 0.007 | 0.001 | 8.66E-09 |  | 0.023 | 0.016 | 1.40E-01 |  | 0.102 | 0.018 | 2.55E-08 |
| rs9810242 | 3 | 46313334 | G | A | 0.016 | 0.002 | 2.22E-12 |  | 0.041 | 0.032 | 1.95E-01 |  | 0.228 | 0.033 | 5.08E-12 |
| rs9838191 | 3 | 46298901 | G | A | 0.018 | 0.003 | 5.39E-12 |  | 0.032 | 0.032 | 3.23E-01 |  | 0.236 | 0.034 | 6.86E-12 |
| rs9838450 | 3 | 46123333 | C | A | 0.016 | 0.002 | 1.04E-20 |  | 0.004 | 0.023 | 8.68E-01 |  | 0.235 | 0.025 | 1.72E-21 |
| rs9845542 | 3 | 46111869 | A | G | 0.016 | 0.002 | 2.21E-21 |  | 0.002 | 0.023 | 9.20E-01 |  | 0.235 | 0.024 | 2.96E-22 |
| rs9872023 | 3 | 46322713 | T | C | 0.016 | 0.002 | 3.72E-12 |  | 0.032 | 0.032 | 3.13E-01 |  | 0.231 | 0.034 | 5.48E-12 |
| rs11085727 | 19 | 10466123 | T | C | 0.008 | 0.001 | 1.42E-08 |  | 0.041 | 0.017 | 1.74E-02 |  | 0.106 | 0.020 | 1.27E-07 |
| rs12610495 | 19 | 4717672 | G | A | 0.008 | 0.001 | 1.89E-08 |  | 0.026 | 0.017 | 1.36E-01 |  | 0.125 | 0.023 | 5.68E-08 |
| rs2109069 | 19 | 4719443 | A | G | 0.010 | 0.001 | 1.06E-14 |  | 0.024 | 0.017 | 1.49E-01 |  | 0.151 | 0.020 | 2.94E-14 |
| rs2277732 | 19 | 4723670 | A | C | 0.010 | 0.001 | 1.03E-13 |  | 0.018 | 0.017 | 2.93E-01 |  | 0.151 | 0.020 | 1.52E-13 |
| rs2304256 | 19 | 10475652 | A | C | 0.008 | 0.001 | 2.96E-08 |  | 0.041 | 0.017 | 1.84E-02 |  | 0.103 | 0.020 | 2.50E-07 |
| rs34725611 | 19 | 10477067 | G | A | 0.008 | 0.001 | 3.74E-08 |  | 0.041 | 0.017 | 1.88E-02 |  | 0.102 | 0.020 | 3.09E-07 |
| rs1051393 | 21 | 34614255 | G | T | 0.010 | 0.001 | 1.32E-13 |  | 0.001 | 0.017 | 9.60E-01 |  | 0.149 | 0.020 | 3.67E-14 |
| rs1131668 | 21 | 34634878 | A | G | 0.008 | 0.001 | 4.14E-10 |  | 0.003 | 0.017 | 8.43E-01 |  | 0.146 | 0.023 | 2.05E-10 |
| rs11911133 | 21 | 34629175 | G | A | 0.008 | 0.001 | 3.94E-10 |  | 0.004 | 0.017 | 8.04E-01 |  | 0.139 | 0.022 | 2.10E-10 |
| rs12053666 | 21 | 34618439 | A | G | 0.009 | 0.001 | 5.17E-12 |  | 0.000 | 0.017 | 9.79E-01 |  | 0.141 | 0.020 | 1.64E-12 |
| rs12482556 | 21 | 34602934 | C | T | 0.010 | 0.001 | 1.14E-14 |  | 0.002 | 0.017 | 8.90E-01 |  | 0.158 | 0.020 | 3.28E-15 |
| rs12627039 | 21 | 34600508 | T | G | 0.009 | 0.001 | 1.10E-11 |  | 0.000 | 0.018 | 9.93E-01 |  | 0.144 | 0.021 | 3.52E-12 |
| rs13050728 | 21 | 34615210 | C | T | -0.011 | 0.001 | 3.16E-16 |  | -0.002 | 0.017 | 9.09E-01 |  | -0.168 | 0.020 | 7.44E-17 |
| rs17860115 | 21 | 34602305 | A | C | 0.010 | 0.001 | 2.89E-14 |  | 0.001 | 0.017 | 9.58E-01 |  | 0.156 | 0.020 | 7.52E-15 |
| rs17860118 | 21 | 34602794 | T | G | 0.014 | 0.002 | 2.83E-09 |  | 0.029 | 0.030 | 3.36E-01 |  | 0.189 | 0.032 | 4.29E-09 |
| rs17860169 | 21 | 34613301 | G | A | 0.010 | 0.001 | 3.07E-14 |  | 0.002 | 0.017 | 9.25E-01 |  | 0.152 | 0.020 | 8.64E-15 |
| rs17860220 | 21 | 34623919 | G | A | 0.014 | 0.002 | 8.46E-10 |  | 0.027 | 0.030 | 3.76E-01 |  | 0.197 | 0.032 | 1.14E-09 |
| rs17860241 | 21 | 34631133 | G | A | 0.009 | 0.001 | 9.89E-11 |  | 0.004 | 0.017 | 8.07E-01 |  | 0.143 | 0.022 | 4.95E-11 |
| rs2073361 | 21 | 34619445 | G | A | -0.011 | 0.001 | 1.07E-15 |  | -0.003 | 0.017 | 8.56E-01 |  | -0.165 | 0.020 | 3.00E-16 |
| rs2073362 | 21 | 34620801 | G | A | 0.014 | 0.002 | 1.70E-09 |  | 0.029 | 0.030 | 3.32E-01 |  | 0.193 | 0.032 | 2.58E-09 |
| rs2229207 | 21 | 34614250 | C | T | 0.014 | 0.002 | 1.95E-09 |  | 0.027 | 0.030 | 3.69E-01 |  | 0.190 | 0.032 | 2.71E-09 |
| rs2236756 | 21 | 34616923 | A | C | -0.011 | 0.001 | 1.06E-15 |  | -0.002 | 0.017 | 9.12E-01 |  | -0.165 | 0.020 | 2.62E-16 |
| rs2236757 | 21 | 34624917 | G | A | -0.010 | 0.001 | 7.00E-14 |  | 0.000 | 0.018 | 9.95E-01 |  | -0.158 | 0.021 | 1.75E-14 |
| rs2236758 | 21 | 34625413 | G | A | -0.009 | 0.001 | 1.45E-10 |  | -0.002 | 0.017 | 9.00E-01 |  | -0.148 | 0.023 | 6.17E-11 |
| rs2250226 | 21 | 34632316 | C | T | 0.009 | 0.001 | 7.06E-11 |  | 0.004 | 0.017 | 8.23E-01 |  | 0.145 | 0.022 | 3.38E-11 |
| rs2252639 | 21 | 34617729 | G | A | -0.011 | 0.001 | 4.26E-16 |  | -0.002 | 0.017 | 8.84E-01 |  | -0.167 | 0.020 | 1.08E-16 |
| rs2284551 | 21 | 34618313 | G | A | -0.010 | 0.001 | 5.29E-14 |  | 0.000 | 0.018 | 9.85E-01 |  | -0.159 | 0.021 | 1.33E-14 |
| rs2300370 | 21 | 34604557 | A | G | 0.010 | 0.001 | 2.48E-14 |  | 0.002 | 0.017 | 8.99E-01 |  | 0.156 | 0.020 | 7.27E-15 |
| rs2834154 | 21 | 34606634 | C | A | 0.010 | 0.001 | 1.88E-14 |  | 0.001 | 0.017 | 9.34E-01 |  | 0.157 | 0.020 | 5.04E-15 |
| rs2834158 | 21 | 34617213 | C | T | -0.009 | 0.001 | 1.02E-10 |  | -0.002 | 0.017 | 9.05E-01 |  | -0.150 | 0.023 | 4.18E-11 |
| rs2834161 | 21 | 34620207 | T | C | -0.011 | 0.001 | 5.15E-16 |  | -0.002 | 0.017 | 8.95E-01 |  | -0.167 | 0.020 | 1.28E-16 |
| rs2834163 | 21 | 34620451 | A | G | -0.011 | 0.001 | 1.04E-15 |  | -0.003 | 0.017 | 8.67E-01 |  | -0.165 | 0.020 | 2.85E-16 |
| rs62226152 | 21 | 34596750 | T | C | 0.009 | 0.001 | 1.38E-11 |  | 0.000 | 0.018 | 9.82E-01 |  | 0.143 | 0.021 | 4.54E-12 |
| rs6517153 | 21 | 34607436 | A | G | 0.010 | 0.001 | 3.42E-14 |  | 0.002 | 0.017 | 8.99E-01 |  | 0.155 | 0.020 | 1.02E-14 |
| rs9636867 | 21 | 34609944 | G | A | 0.010 | 0.001 | 2.33E-14 |  | 0.003 | 0.017 | 8.68E-01 |  | 0.151 | 0.019 | 7.32E-15 |
| rs9975448 | 21 | 34626855 | G | A | -0.008 | 0.001 | 2.70E-08 |  | -0.003 | 0.018 | 8.76E-01 |  | -0.133 | 0.024 | 1.50E-08 |
| rs9975538 | 21 | 34626854 | T | C | -0.009 | 0.001 | 8.50E-11 |  | -0.005 | 0.017 | 7.69E-01 |  | -0.149 | 0.023 | 4.54E-11 |
| rs9976829 | 21 | 34614834 | A | G | -0.011 | 0.001 | 5.49E-16 |  | -0.002 | 0.017 | 9.23E-01 |  | -0.167 | 0.020 | 1.28E-16 |
| SNP, single nucleotide polymorphism; A1, effect allele; A2, reference allele. | | | | | | | | | | | | | | | |

**Table S4.** Significant genetic variants associated with cross-trait osteoarthritis and COVID-19 hospitalization.

| **SNP** | **Chr** | **Position** | **A1** | **A2** | **Beta_mtag_** | **SE_meta_** | *P*_meta_ |  | **Osteoarthritis** | | |  | **COVID-19 hospitalization** | | |
| --- | --- | --- | --- | --- | --- | --- | --- | --- | --- | --- | --- | --- | --- | --- | --- |
|  |  |  |  |  |  |  |  |  | **Beta** | **SE** | ***P*_value** |  | **Beta** | **SE** | ***P*_value** |
| rs10510748 | 3 | 46178538 | G | A | 0.017 | 0.003 | 9.11E-10 |  | 0.031 | 0.029 | 2.89E-01 |  | 0.271 | 0.044 | 1.13E-09 |
| rs10510749 | 3 | 46180416 | T | C | 0.018 | 0.003 | 6.88E-10 |  | 0.024 | 0.030 | 4.18E-01 |  | 0.285 | 0.046 | 5.26E-10 |
| rs10510750 | 3 | 46274886 | C | T | 0.018 | 0.003 | 3.49E-09 |  | 0.018 | 0.032 | 5.83E-01 |  | 0.295 | 0.049 | 1.79E-09 |
| rs114115904 | 3 | 46268664 | T | C | 0.019 | 0.003 | 6.12E-10 |  | 0.006 | 0.032 | 8.52E-01 |  | 0.306 | 0.048 | 1.40E-10 |
| rs115102354 | 3 | 46222037 | G | A | 0.028 | 0.004 | 1.35E-14 |  | 0.020 | 0.038 | 5.99E-01 |  | 0.441 | 0.056 | 2.83E-15 |
| rs11926063 | 3 | 46263717 | G | A | 0.017 | 0.003 | 3.81E-08 |  | 0.012 | 0.031 | 6.97E-01 |  | 0.250 | 0.044 | 1.74E-08 |
| rs12108042 | 3 | 46086083 | G | A | 0.023 | 0.002 | 3.88E-21 |  | 0.001 | 0.025 | 9.83E-01 |  | 0.363 | 0.037 | 6.03E-23 |
| rs13059906 | 3 | 46208858 | C | T | 0.017 | 0.003 | 8.88E-09 |  | 0.032 | 0.030 | 2.87E-01 |  | 0.256 | 0.045 | 1.21E-08 |
| rs13060713 | 3 | 46272709 | C | A | 0.019 | 0.003 | 3.30E-10 |  | 0.006 | 0.032 | 8.48E-01 |  | 0.311 | 0.048 | 7.21E-11 |
| rs13061548 | 3 | 46226646 | C | T | 0.016 | 0.003 | 1.92E-08 |  | 0.034 | 0.029 | 2.46E-01 |  | 0.239 | 0.043 | 3.16E-08 |
| rs13062450 | 3 | 46227171 | G | T | 0.017 | 0.003 | 3.43E-09 |  | 0.028 | 0.029 | 3.43E-01 |  | 0.259 | 0.044 | 3.65E-09 |
| rs13065041 | 3 | 46289403 | T | G | 0.018 | 0.003 | 7.16E-09 |  | 0.027 | 0.032 | 4.05E-01 |  | 0.290 | 0.050 | 6.42E-09 |
| rs13065351 | 3 | 46327512 | G | T | 0.017 | 0.003 | 1.21E-08 |  | 0.029 | 0.032 | 3.67E-01 |  | 0.282 | 0.050 | 1.26E-08 |
| rs13066062 | 3 | 46018344 | A | G | 0.027 | 0.003 | 4.25E-25 |  | 0.007 | 0.027 | 7.88E-01 |  | 0.424 | 0.039 | 6.31E-27 |
| rs13066516 | 3 | 45975443 | T | C | 0.027 | 0.003 | 8.64E-25 |  | 0.006 | 0.027 | 8.30E-01 |  | 0.424 | 0.040 | 1.15E-26 |
| rs13067058 | 3 | 46273766 | A | G | 0.018 | 0.003 | 3.89E-09 |  | 0.008 | 0.032 | 7.97E-01 |  | 0.299 | 0.049 | 1.18E-09 |
| rs13068145 | 3 | 46332276 | A | C | 0.017 | 0.003 | 1.91E-08 |  | 0.029 | 0.032 | 3.69E-01 |  | 0.281 | 0.050 | 2.01E-08 |
| rs13068271 | 3 | 46332184 | A | G | 0.017 | 0.003 | 1.93E-08 |  | 0.029 | 0.032 | 3.66E-01 |  | 0.281 | 0.050 | 2.05E-08 |
| rs13069079 | 3 | 46000870 | A | G | 0.027 | 0.003 | 1.48E-24 |  | 0.004 | 0.027 | 8.70E-01 |  | 0.421 | 0.040 | 1.75E-26 |
| rs13069750 | 3 | 46224850 | T | C | 0.018 | 0.003 | 6.41E-10 |  | 0.023 | 0.030 | 4.40E-01 |  | 0.287 | 0.046 | 4.54E-10 |
| rs13069845 | 3 | 46232132 | T | C | 0.018 | 0.003 | 6.87E-10 |  | 0.024 | 0.030 | 4.20E-01 |  | 0.286 | 0.046 | 5.21E-10 |
| rs13070099 | 3 | 46253214 | C | T | 0.018 | 0.003 | 1.88E-09 |  | 0.018 | 0.030 | 5.45E-01 |  | 0.283 | 0.046 | 1.03E-09 |
| rs13071283 | 3 | 46010007 | C | T | 0.027 | 0.003 | 5.82E-25 |  | 0.014 | 0.026 | 5.87E-01 |  | 0.417 | 0.039 | 2.14E-26 |
| rs13071469 | 3 | 46328627 | C | T | 0.018 | 0.003 | 1.15E-08 |  | 0.029 | 0.032 | 3.73E-01 |  | 0.283 | 0.050 | 1.16E-08 |
| rs13073976 | 3 | 46286663 | C | T | 0.018 | 0.003 | 2.43E-09 |  | 0.027 | 0.032 | 4.06E-01 |  | 0.296 | 0.049 | 2.06E-09 |
| rs13075270 | 3 | 46253789 | C | T | 0.016 | 0.003 | 1.37E-08 |  | 0.025 | 0.029 | 3.87E-01 |  | 0.248 | 0.044 | 1.34E-08 |
| rs13075758 | 3 | 46025048 | A | G | 0.027 | 0.003 | 7.39E-25 |  | 0.008 | 0.027 | 7.77E-01 |  | 0.422 | 0.039 | 1.20E-26 |
| rs13075836 | 3 | 46186638 | C | T | 0.018 | 0.003 | 4.56E-10 |  | 0.024 | 0.030 | 4.26E-01 |  | 0.290 | 0.046 | 3.31E-10 |
| rs13077302 | 3 | 46329475 | C | T | 0.017 | 0.003 | 1.23E-08 |  | 0.029 | 0.032 | 3.67E-01 |  | 0.282 | 0.050 | 1.28E-08 |
| rs13078564 | 3 | 46330205 | C | T | 0.017 | 0.003 | 1.44E-08 |  | 0.026 | 0.032 | 4.20E-01 |  | 0.282 | 0.050 | 1.28E-08 |
| rs13078739 | 3 | 46007488 | A | G | 0.027 | 0.003 | 8.51E-25 |  | 0.011 | 0.027 | 6.72E-01 |  | 0.417 | 0.039 | 2.20E-26 |
| rs13079478 | 3 | 46007823 | T | G | 0.028 | 0.003 | 1.09E-25 |  | 0.005 | 0.027 | 8.54E-01 |  | 0.431 | 0.040 | 1.09E-27 |
| rs13079869 | 3 | 46008087 | A | G | 0.027 | 0.003 | 3.93E-25 |  | 0.008 | 0.027 | 7.69E-01 |  | 0.424 | 0.039 | 6.26E-27 |
| rs13080979 | 3 | 46233690 | G | A | 0.018 | 0.003 | 8.58E-10 |  | 0.024 | 0.030 | 4.20E-01 |  | 0.285 | 0.046 | 6.58E-10 |
| rs13081151 | 3 | 46055716 | A | G | 0.027 | 0.003 | 5.95E-24 |  | 0.007 | 0.027 | 7.93E-01 |  | 0.422 | 0.040 | 1.08E-25 |
| rs13082995 | 3 | 46242477 | C | T | 0.017 | 0.003 | 4.35E-09 |  | 0.027 | 0.029 | 3.52E-01 |  | 0.258 | 0.044 | 4.54E-09 |
| rs13083914 | 3 | 46184680 | T | C | 0.017 | 0.003 | 2.01E-09 |  | 0.023 | 0.030 | 4.37E-01 |  | 0.277 | 0.046 | 1.53E-09 |
| rs13086063 | 3 | 46200860 | C | T | 0.016 | 0.003 | 8.36E-09 |  | 0.032 | 0.029 | 2.78E-01 |  | 0.255 | 0.045 | 1.18E-08 |
| rs13089543 | 3 | 46183560 | G | T | 0.017 | 0.003 | 2.78E-09 |  | 0.029 | 0.029 | 3.22E-01 |  | 0.264 | 0.045 | 3.17E-09 |
| rs13089544 | 3 | 46183561 | C | T | 0.017 | 0.003 | 4.54E-09 |  | 0.029 | 0.029 | 3.22E-01 |  | 0.261 | 0.045 | 5.29E-09 |
| rs13089907 | 3 | 46215710 | T | C | 0.017 | 0.003 | 6.83E-09 |  | 0.032 | 0.030 | 2.81E-01 |  | 0.258 | 0.045 | 9.45E-09 |
| rs13092160 | 3 | 46254791 | C | T | 0.017 | 0.003 | 2.39E-09 |  | 0.020 | 0.030 | 5.10E-01 |  | 0.276 | 0.046 | 1.47E-09 |
| rs13093063 | 3 | 46329368 | T | C | 0.017 | 0.003 | 1.21E-08 |  | 0.029 | 0.032 | 3.67E-01 |  | 0.282 | 0.050 | 1.26E-08 |
| rs13093179 | 3 | 46146314 | T | G | 0.016 | 0.003 | 3.60E-09 |  | 0.000 | 0.027 | 9.95E-01 |  | 0.328 | 0.053 | 6.62E-10 |
| rs13095940 | 3 | 46246816 | G | A | 0.018 | 0.003 | 1.53E-09 |  | 0.020 | 0.03 | 4.97E-01 |  | 0.283 | 0.046 | 9.51E-10 |
| rs13095946 | 3 | 46253812 | A | G | 0.017 | 0.003 | 2.19E-09 |  | 0.019 | 0.030 | 5.26E-01 |  | 0.281 | 0.046 | 1.29E-09 |
| rs13096307 | 3 | 46246953 | T | C | 0.018 | 0.003 | 1.53E-09 |  | 0.020 | 0.030 | 4.97E-01 |  | 0.283 | 0.046 | 9.55E-10 |
| rs13096325 | 3 | 46186838 | G | A | 0.018 | 0.003 | 5.62E-10 |  | 0.024 | 0.030 | 4.30E-01 |  | 0.288 | 0.046 | 4.08E-10 |
| rs13096808 | 3 | 46274906 | T | C | 0.018 | 0.003 | 4.47E-09 |  | 0.017 | 0.032 | 6.05E-01 |  | 0.295 | 0.049 | 2.21E-09 |
| rs13096905 | 3 | 46274766 | A | G | 0.018 | 0.003 | 4.27E-09 |  | 0.017 | 0.032 | 6.05E-01 |  | 0.295 | 0.049 | 2.10E-09 |
| rs13097340 | 3 | 46187118 | A | G | 0.018 | 0.003 | 4.84E-10 |  | 0.024 | 0.030 | 4.30E-01 |  | 0.289 | 0.046 | 3.49E-10 |
| rs13097666 | 3 | 46234467 | T | C | 0.017 | 0.003 | 9.92E-10 |  | 0.024 | 0.030 | 4.15E-01 |  | 0.283 | 0.046 | 7.80E-10 |
| rs13098911 | 3 | 46235201 | T | C | 0.018 | 0.003 | 5.60E-10 |  | 0.025 | 0.030 | 3.98E-01 |  | 0.287 | 0.046 | 4.52E-10 |
| rs13325613 | 3 | 46298373 | T | G | 0.018 | 0.003 | 7.42E-09 |  | 0.039 | 0.032 | 2.25E-01 |  | 0.275 | 0.048 | 1.31E-08 |
| rs13433997 | 3 | 46049765 | C | T | 0.026 | 0.003 | 7.42E-24 |  | 0.020 | 0.027 | 4.48E-01 |  | 0.397 | 0.039 | 6.45E-25 |
| rs138940209 | 3 | 46371581 | A | G | 0.017 | 0.003 | 2.99E-08 |  | 0.039 | 0.032 | 2.28E-01 |  | 0.274 | 0.050 | 5.40E-08 |
| rs1392290 | 3 | 46069210 | A | G | 0.010 | 0.002 | 2.06E-09 |  | 0.000 | 0.017 | 9.95E-01 |  | 0.172 | 0.027 | 3.69E-10 |
| rs140295517 | 3 | 46368690 | G | A | 0.017 | 0.003 | 3.75E-08 |  | 0.034 | 0.032 | 2.87E-01 |  | 0.274 | 0.050 | 5.40E-08 |
| rs142039684 | 3 | 46371793 | C | T | 0.017 | 0.003 | 4.97E-08 |  | 0.035 | 0.032 | 2.79E-01 |  | 0.271 | 0.050 | 7.43E-08 |
| rs146375688 | 3 | 46388700 | T | C | 0.026 | 0.004 | 6.82E-13 |  | 0.043 | 0.039 | 2.71E-01 |  | 0.425 | 0.059 | 6.72E-13 |
| rs1491951 | 3 | 46141844 | A | G | 0.024 | 0.003 | 1.82E-20 |  | 0.002 | 0.027 | 9.44E-01 |  | 0.384 | 0.040 | 3.74E-22 |
| rs1542755 | 3 | 46272440 | T | G | 0.020 | 0.003 | 2.91E-10 |  | 0.008 | 0.032 | 8.12E-01 |  | 0.311 | 0.048 | 6.92E-11 |
| rs1542756 | 3 | 46272162 | T | G | 0.021 | 0.003 | 1.75E-11 |  | 0.016 | 0.032 | 6.14E-01 |  | 0.325 | 0.047 | 5.80E-12 |
| rs17214952 | 3 | 46011436 | G | A | 0.027 | 0.003 | 2.98E-25 |  | 0.013 | 0.026 | 6.17E-01 |  | 0.419 | 0.039 | 9.05E-27 |
| rs17215008 | 3 | 46012279 | C | T | 0.027 | 0.003 | 5.65E-25 |  | 0.008 | 0.027 | 7.72E-01 |  | 0.423 | 0.039 | 9.20E-27 |
| rs17216717 | 3 | 46186323 | C | T | 0.018 | 0.003 | 4.97E-10 |  | 0.023 | 0.030 | 4.35E-01 |  | 0.289 | 0.046 | 3.53E-10 |
| rs17217831 | 3 | 46305441 | A | C | 0.017 | 0.003 | 1.22E-08 |  | 0.035 | 0.032 | 2.70E-01 |  | 0.277 | 0.049 | 1.81E-08 |
| rs17282391 | 3 | 46179481 | G | A | 0.018 | 0.003 | 1.45E-10 |  | 0.024 | 0.030 | 4.30E-01 |  | 0.299 | 0.046 | 9.75E-11 |
| rs17282797 | 3 | 46232765 | G | A | 0.018 | 0.003 | 8.60E-10 |  | 0.024 | 0.030 | 4.20E-01 |  | 0.285 | 0.046 | 6.60E-10 |
| rs17282922 | 3 | 46240172 | G | T | 0.018 | 0.003 | 9.84E-10 |  | 0.022 | 0.030 | 4.71E-01 |  | 0.285 | 0.046 | 6.47E-10 |
| rs17283712 | 3 | 46304064 | G | T | 0.018 | 0.003 | 9.35E-09 |  | 0.027 | 0.032 | 4.04E-01 |  | 0.285 | 0.049 | 8.52E-09 |
| rs17284138 | 3 | 46331852 | C | A | 0.017 | 0.003 | 2.11E-08 |  | 0.029 | 0.032 | 3.67E-01 |  | 0.28 | 0.050 | 2.25E-08 |
| rs17330872 | 3 | 46035097 | G | A | 0.027 | 0.003 | 8.92E-25 |  | 0.008 | 0.027 | 7.55E-01 |  | 0.421 | 0.040 | 1.62E-26 |
| rs1994492 | 3 | 45960646 | C | T | 0.027 | 0.003 | 1.79E-25 |  | 0.009 | 0.027 | 7.48E-01 |  | 0.431 | 0.040 | 2.93E-27 |
| rs1994493 | 3 | 45960700 | T | C | 0.027 | 0.003 | 2.31E-25 |  | 0.006 | 0.027 | 8.17E-01 |  | 0.431 | 0.040 | 2.88E-27 |
| rs2171531 | 3 | 45981171 | T | C | 0.027 | 0.003 | 1.58E-24 |  | 0.004 | 0.027 | 8.83E-01 |  | 0.421 | 0.040 | 1.77E-26 |
| rs2373087 | 3 | 45968043 | G | T | 0.027 | 0.003 | 1.58E-24 |  | 0.003 | 0.027 | 9.02E-01 |  | 0.423 | 0.040 | 1.65E-26 |
| rs28677778 | 3 | 46322171 | G | A | 0.017 | 0.003 | 1.76E-08 |  | 0.039 | 0.032 | 2.21E-01 |  | 0.267 | 0.048 | 3.24E-08 |
| rs3091314 | 3 | 46188753 | A | G | 0.018 | 0.003 | 4.40E-10 |  | 0.024 | 0.030 | 4.17E-01 |  | 0.289 | 0.046 | 3.30E-10 |
| rs3092957 | 3 | 46403961 | A | G | 0.017 | 0.003 | 4.57E-08 |  | 0.037 | 0.032 | 2.53E-01 |  | 0.271 | 0.050 | 7.54E-08 |
| rs3092959 | 3 | 46403468 | A | G | 0.017 | 0.003 | 4.99E-08 |  | 0.039 | 0.032 | 2.23E-01 |  | 0.269 | 0.050 | 9.35E-08 |
| rs3136672 | 3 | 46242785 | C | T | 0.015 | 0.003 | 3.86E-08 |  | 0.031 | 0.029 | 2.88E-01 |  | 0.234 | 0.043 | 5.53E-08 |
| rs3136673 | 3 | 46242616 | T | C | 0.017 | 0.003 | 4.19E-09 |  | 0.028 | 0.029 | 3.43E-01 |  | 0.258 | 0.044 | 4.51E-09 |
| rs3176824 | 3 | 46248385 | C | T | 0.018 | 0.003 | 1.55E-09 |  | 0.020 | 0.030 | 4.98E-01 |  | 0.283 | 0.046 | 9.67E-10 |
| rs3176825 | 3 | 46248224 | T | G | 0.018 | 0.003 | 1.52E-09 |  | 0.020 | 0.030 | 4.98E-01 |  | 0.283 | 0.046 | 9.48E-10 |
| rs3176826 | 3 | 46247584 | A | G | 0.018 | 0.003 | 1.54E-09 |  | 0.021 | 0.030 | 4.88E-01 |  | 0.283 | 0.046 | 9.87E-10 |
| rs3181076 | 3 | 46250733 | T | C | 0.018 | 0.003 | 1.71E-09 |  | 0.020 | 0.030 | 5.10E-01 |  | 0.283 | 0.046 | 1.03E-09 |
| rs3181078 | 3 | 46250584 | C | A | 0.018 | 0.003 | 1.77E-09 |  | 0.020 | 0.030 | 5.01E-01 |  | 0.282 | 0.046 | 1.10E-09 |
| rs33910087 | 3 | 46009487 | A | G | 0.027 | 0.003 | 6.16E-25 |  | 0.009 | 0.027 | 7.23E-01 |  | 0.421 | 0.039 | 1.24E-26 |
| rs33998492 | 3 | 46273598 | A | G | 0.018 | 0.003 | 3.68E-09 |  | 0.006 | 0.032 | 8.49E-01 |  | 0.300 | 0.049 | 9.80E-10 |
| rs34000569 | 3 | 45999209 | G | A | 0.027 | 0.003 | 1.23E-24 |  | 0.006 | 0.027 | 8.17E-01 |  | 0.421 | 0.040 | 1.78E-26 |
| rs34005848 | 3 | 46263745 | T | C | 0.019 | 0.003 | 5.29E-10 |  | 0.007 | 0.032 | 8.35E-01 |  | 0.307 | 0.048 | 1.25E-10 |
| rs34013035 | 3 | 46220682 | A | G | 0.017 | 0.003 | 5.65E-09 |  | 0.033 | 0.030 | 2.71E-01 |  | 0.258 | 0.045 | 8.06E-09 |
| rs34059564 | 3 | 46226165 | C | T | 0.016 | 0.003 | 1.73E-08 |  | 0.035 | 0.029 | 2.32E-01 |  | 0.239 | 0.043 | 3.02E-08 |
| rs34068335 | 3 | 45954339 | T | C | 0.028 | 0.003 | 1.12E-25 |  | 0.003 | 0.027 | 9.23E-01 |  | 0.443 | 0.041 | 8.44E-28 |
| rs34073838 | 3 | 46337692 | C | A | 0.017 | 0.003 | 2.26E-08 |  | 0.027 | 0.032 | 3.98E-01 |  | 0.280 | 0.050 | 2.19E-08 |
| rs34079287 | 3 | 46300870 | T | G | 0.018 | 0.003 | 9.21E-09 |  | 0.027 | 0.032 | 4.04E-01 |  | 0.285 | 0.049 | 8.38E-09 |
| rs34093271 | 3 | 46144981 | T | G | 0.016 | 0.003 | 3.43E-09 |  | 0.001 | 0.027 | 9.84E-01 |  | 0.329 | 0.053 | 6.45E-10 |
| rs34101673 | 3 | 46197286 | T | C | 0.018 | 0.003 | 9.54E-10 |  | 0.024 | 0.030 | 4.26E-01 |  | 0.285 | 0.046 | 7.21E-10 |
| rs34127208 | 3 | 46103680 | T | G | 0.025 | 0.003 | 3.98E-22 |  | 0.002 | 0.027 | 9.42E-01 |  | 0.407 | 0.040 | 5.85E-24 |
| rs34134191 | 3 | 46221531 | C | T | 0.018 | 0.003 | 6.46E-10 |  | 0.024 | 0.030 | 4.17E-01 |  | 0.287 | 0.046 | 4.93E-10 |
| rs34155121 | 3 | 46084742 | A | G | 0.023 | 0.002 | 4.21E-21 |  | 0.001 | 0.025 | 9.81E-01 |  | 0.362 | 0.037 | 6.60E-23 |
| rs34168660 | 3 | 46056162 | A | G | 0.027 | 0.003 | 1.90E-24 |  | 0.007 | 0.027 | 8.05E-01 |  | 0.412 | 0.039 | 2.98E-26 |
| rs34180919 | 3 | 46353199 | A | G | 0.017 | 0.003 | 3.84E-08 |  | 0.034 | 0.032 | 2.88E-01 |  | 0.273 | 0.050 | 5.50E-08 |
| rs34191675 | 3 | 46189448 | G | A | 0.017 | 0.003 | 1.36E-09 |  | 0.025 | 0.030 | 4.05E-01 |  | 0.277 | 0.046 | 1.13E-09 |
| rs34194160 | 3 | 46265854 | A | G | 0.019 | 0.003 | 5.75E-10 |  | 0.006 | 0.032 | 8.48E-01 |  | 0.306 | 0.048 | 1.32E-10 |
| rs34198655 | 3 | 46215107 | A | G | 0.017 | 0.003 | 7.69E-09 |  | 0.031 | 0.030 | 2.95E-01 |  | 0.257 | 0.045 | 1.01E-08 |
| rs34272024 | 3 | 46292339 | A | G | 0.017 | 0.003 | 3.60E-08 |  | 0.038 | 0.031 | 2.28E-01 |  | 0.253 | 0.047 | 6.55E-08 |
| rs34289272 | 3 | 46263244 | T | C | 0.019 | 0.003 | 3.82E-10 |  | 0.010 | 0.032 | 7.48E-01 |  | 0.308 | 0.048 | 1.09E-10 |
| rs34324101 | 3 | 46000728 | G | T | 0.027 | 0.003 | 1.54E-24 |  | 0.004 | 0.027 | 8.76E-01 |  | 0.421 | 0.040 | 1.78E-26 |
| rs34340501 | 3 | 46225216 | T | G | 0.018 | 0.003 | 6.51E-10 |  | 0.023 | 0.030 | 4.39E-01 |  | 0.287 | 0.046 | 4.62E-10 |
| rs34340587 | 3 | 46096043 | A | G | 0.016 | 0.003 | 1.28E-09 |  | 0.000 | 0.027 | 9.93E-01 |  | 0.336 | 0.053 | 2.16E-10 |
| rs34351442 | 3 | 46355492 | A | G | 0.017 | 0.003 | 3.92E-08 |  | 0.034 | 0.032 | 2.95E-01 |  | 0.273 | 0.050 | 5.48E-08 |
| rs34378541 | 3 | 46239649 | T | C | 0.018 | 0.003 | 9.03E-10 |  | 0.023 | 0.030 | 4.40E-01 |  | 0.285 | 0.046 | 6.51E-10 |
| rs34381952 | 3 | 45995748 | C | T | 0.026 | 0.003 | 2.57E-24 |  | 0.009 | 0.026 | 7.29E-01 |  | 0.414 | 0.039 | 5.71E-26 |
| rs34386754 | 3 | 46090013 | A | G | 0.014 | 0.002 | 4.08E-08 |  | 0.001 | 0.025 | 9.62E-01 |  | 0.272 | 0.047 | 1.01E-08 |
| rs34401473 | 3 | 46189772 | C | A | 0.017 | 0.003 | 1.75E-09 |  | 0.025 | 0.030 | 4.05E-01 |  | 0.276 | 0.046 | 1.47E-09 |
| rs34406035 | 3 | 46278919 | T | C | 0.018 | 0.003 | 3.25E-09 |  | 0.024 | 0.032 | 4.64E-01 |  | 0.295 | 0.049 | 2.34E-09 |
| rs34409248 | 3 | 46198071 | A | G | 0.018 | 0.003 | 9.53E-10 |  | 0.024 | 0.030 | 4.26E-01 |  | 0.285 | 0.046 | 7.21E-10 |
| rs34414382 | 3 | 46265190 | T | C | 0.019 | 0.003 | 5.57E-10 |  | 0.006 | 0.032 | 8.48E-01 |  | 0.307 | 0.048 | 1.27E-10 |
| rs34423195 | 3 | 46249722 | G | A | 0.018 | 0.003 | 1.59E-09 |  | 0.020 | 0.030 | 4.98E-01 |  | 0.283 | 0.046 | 9.90E-10 |
| rs34452002 | 3 | 46143187 | T | C | 0.016 | 0.003 | 3.45E-09 |  | 0.000 | 0.027 | 9.93E-01 |  | 0.329 | 0.053 | 6.35E-10 |
| rs34460587 | 3 | 46142464 | T | C | 0.025 | 0.003 | 1.30E-21 |  | 0.000 | 0.027 | 9.87E-01 |  | 0.403 | 0.040 | 1.79E-23 |
| rs34493660 | 3 | 46052800 | A | G | 0.026 | 0.003 | 4.31E-24 |  | 0.010 | 0.027 | 7.19E-01 |  | 0.407 | 0.039 | 1.04E-25 |
| rs34523728 | 3 | 46219833 | A | C | 0.018 | 0.003 | 5.96E-10 |  | 0.024 | 0.030 | 4.17E-01 |  | 0.287 | 0.046 | 4.52E-10 |
| rs34531115 | 3 | 46257696 | C | T | 0.018 | 0.003 | 1.83E-09 |  | 0.019 | 0.030 | 5.37E-01 |  | 0.283 | 0.046 | 1.03E-09 |
| rs34558763 | 3 | 46189924 | T | C | 0.018 | 0.003 | 4.39E-10 |  | 0.024 | 0.030 | 4.17E-01 |  | 0.289 | 0.046 | 3.28E-10 |
| rs34567015 | 3 | 46202212 | A | G | 0.018 | 0.003 | 9.26E-10 |  | 0.025 | 0.030 | 4.11E-01 |  | 0.284 | 0.046 | 7.33E-10 |
| rs34570200 | 3 | 46220500 | T | C | 0.018 | 0.003 | 5.90E-10 |  | 0.024 | 0.030 | 4.17E-01 |  | 0.288 | 0.046 | 4.48E-10 |
| rs34677490 | 3 | 46270748 | T | G | 0.019 | 0.003 | 6.10E-10 |  | 0.006 | 0.032 | 8.52E-01 |  | 0.306 | 0.048 | 1.39E-10 |
| rs34679077 | 3 | 46087992 | A | G | 0.026 | 0.003 | 1.79E-22 |  | 0.000 | 0.027 | 9.95E-01 |  | 0.410 | 0.040 | 2.00E-24 |
| rs34692251 | 3 | 46273727 | A | G | 0.018 | 0.003 | 7.07E-09 |  | 0.008 | 0.032 | 7.91E-01 |  | 0.294 | 0.049 | 2.28E-09 |
| rs34693386 | 3 | 46195402 | T | C | 0.018 | 0.003 | 5.62E-10 |  | 0.024 | 0.030 | 4.35E-01 |  | 0.289 | 0.046 | 4.01E-10 |
| rs34745455 | 3 | 46267333 | C | T | 0.019 | 0.003 | 5.95E-10 |  | 0.006 | 0.032 | 8.52E-01 |  | 0.306 | 0.048 | 1.36E-10 |
| rs34754340 | 3 | 46041837 | T | C | 0.027 | 0.003 | 2.30E-24 |  | 0.010 | 0.027 | 7.17E-01 |  | 0.417 | 0.040 | 5.30E-26 |
| rs34766614 | 3 | 46107601 | G | A | 0.025 | 0.003 | 3.50E-22 |  | 0.001 | 0.027 | 9.68E-01 |  | 0.408 | 0.040 | 4.59E-24 |
| rs34836513 | 3 | 46046459 | A | G | 0.027 | 0.003 | 1.87E-24 |  | 0.010 | 0.027 | 7.01E-01 |  | 0.417 | 0.040 | 4.54E-26 |
| rs34849862 | 3 | 46001367 | A | C | 0.027 | 0.003 | 1.48E-24 |  | 0.004 | 0.027 | 8.70E-01 |  | 0.421 | 0.040 | 1.74E-26 |
| rs34865316 | 3 | 46247456 | G | A | 0.018 | 0.003 | 1.53E-09 |  | 0.020 | 0.030 | 4.97E-01 |  | 0.283 | 0.046 | 9.57E-10 |
| rs34867672 | 3 | 46088282 | C | T | 0.023 | 0.002 | 4.05E-21 |  | 0.001 | 0.025 | 9.62E-01 |  | 0.362 | 0.037 | 6.84E-23 |
| rs34870159 | 3 | 46189490 | C | T | 0.017 | 0.003 | 1.39E-09 |  | 0.024 | 0.030 | 4.22E-01 |  | 0.278 | 0.046 | 1.09E-09 |
| rs34897745 | 3 | 46198376 | C | A | 0.018 | 0.003 | 1.11E-09 |  | 0.021 | 0.030 | 4.77E-01 |  | 0.285 | 0.046 | 7.23E-10 |
| rs34919616 | 3 | 46250008 | A | G | 0.018 | 0.003 | 1.34E-09 |  | 0.023 | 0.030 | 4.39E-01 |  | 0.283 | 0.046 | 9.91E-10 |
| rs34920132 | 3 | 46226769 | G | A | 0.016 | 0.003 | 2.00E-08 |  | 0.034 | 0.029 | 2.47E-01 |  | 0.239 | 0.043 | 3.30E-08 |
| rs34924300 | 3 | 46088336 | T | G | 0.023 | 0.002 | 4.06E-21 |  | 0.001 | 0.025 | 9.62E-01 |  | 0.362 | 0.037 | 6.86E-23 |
| rs34988015 | 3 | 46356048 | C | T | 0.017 | 0.003 | 3.63E-08 |  | 0.034 | 0.032 | 2.97E-01 |  | 0.274 | 0.050 | 5.03E-08 |
| rs35035328 | 3 | 46276078 | C | T | 0.018 | 0.003 | 4.34E-09 |  | 0.019 | 0.032 | 5.61E-01 |  | 0.293 | 0.049 | 2.41E-09 |
| rs35110864 | 3 | 46154457 | A | G | 0.016 | 0.003 | 3.93E-09 |  | 0.001 | 0.027 | 9.77E-01 |  | 0.327 | 0.053 | 7.62E-10 |
| rs35117954 | 3 | 46338831 | G | A | 0.017 | 0.003 | 1.61E-08 |  | 0.029 | 0.032 | 3.76E-01 |  | 0.283 | 0.050 | 1.65E-08 |
| rs35161099 | 3 | 46067507 | G | T | 0.026 | 0.003 | 1.00E-22 |  | 0.001 | 0.027 | 9.85E-01 |  | 0.407 | 0.040 | 1.10E-24 |
| rs35162796 | 3 | 46204379 | T | C | 0.018 | 0.003 | 9.58E-10 |  | 0.024 | 0.030 | 4.17E-01 |  | 0.285 | 0.046 | 7.48E-10 |
| rs35203745 | 3 | 46322289 | C | T | 0.017 | 0.003 | 3.15E-08 |  | 0.040 | 0.032 | 2.07E-01 |  | 0.260 | 0.048 | 6.26E-08 |
| rs35209528 | 3 | 46003496 | C | T | 0.027 | 0.003 | 1.46E-24 |  | 0.004 | 0.027 | 8.70E-01 |  | 0.421 | 0.040 | 1.72E-26 |
| rs35218998 | 3 | 46328715 | C | T | 0.017 | 0.003 | 1.24E-08 |  | 0.029 | 0.032 | 3.67E-01 |  | 0.282 | 0.050 | 1.28E-08 |
| rs35280891 | 3 | 45951647 | A | G | 0.017 | 0.003 | 1.17E-10 |  | 0.005 | 0.027 | 8.55E-01 |  | 0.357 | 0.053 | 2.24E-11 |
| rs35354367 | 3 | 46250799 | G | A | 0.018 | 0.003 | 1.74E-09 |  | 0.020 | 0.030 | 5.11E-01 |  | 0.283 | 0.046 | 1.05E-09 |
| rs35373513 | 3 | 46312953 | T | C | 0.018 | 0.003 | 9.67E-09 |  | 0.029 | 0.032 | 3.63E-01 |  | 0.284 | 0.049 | 1.01E-08 |
| rs35429781 | 3 | 46253607 | C | A | 0.018 | 0.003 | 1.70E-09 |  | 0.020 | 0.030 | 5.10E-01 |  | 0.283 | 0.046 | 1.03E-09 |
| rs35434266 | 3 | 46206999 | T | G | 0.018 | 0.003 | 9.41E-10 |  | 0.024 | 0.030 | 4.32E-01 |  | 0.285 | 0.046 | 6.98E-10 |
| rs35477280 | 3 | 45974092 | A | G | 0.027 | 0.003 | 7.27E-25 |  | 0.006 | 0.027 | 8.18E-01 |  | 0.425 | 0.040 | 9.96E-27 |
| rs35481399 | 3 | 46265580 | A | C | 0.017 | 0.003 | 4.23E-08 |  | 0.011 | 0.031 | 7.09E-01 |  | 0.251 | 0.045 | 1.89E-08 |
| rs35501575 | 3 | 45993645 | T | C | 0.027 | 0.003 | 1.37E-24 |  | 0.005 | 0.027 | 8.40E-01 |  | 0.421 | 0.040 | 1.82E-26 |
| rs35511592 | 3 | 46334846 | A | G | 0.017 | 0.003 | 1.97E-08 |  | 0.029 | 0.032 | 3.76E-01 |  | 0.281 | 0.050 | 2.03E-08 |
| rs35516580 | 3 | 46107498 | G | A | 0.026 | 0.003 | 1.74E-22 |  | 0.001 | 0.027 | 9.71E-01 |  | 0.411 | 0.040 | 2.13E-24 |
| rs35525815 | 3 | 46006269 | T | C | 0.027 | 0.003 | 1.01E-24 |  | 0.011 | 0.027 | 6.72E-01 |  | 0.416 | 0.039 | 2.65E-26 |
| rs35539222 | 3 | 46154272 | C | T | 0.016 | 0.003 | 3.93E-09 |  | 0.001 | 0.027 | 9.77E-01 |  | 0.327 | 0.053 | 7.61E-10 |
| rs35560301 | 3 | 46220506 | G | T | 0.018 | 0.003 | 5.94E-10 |  | 0.024 | 0.030 | 4.17E-01 |  | 0.288 | 0.046 | 4.51E-10 |
| rs35566550 | 3 | 46210512 | T | G | 0.018 | 0.003 | 8.75E-10 |  | 0.023 | 0.030 | 4.35E-01 |  | 0.286 | 0.046 | 6.40E-10 |
| rs35587265 | 3 | 46259296 | G | A | 0.018 | 0.003 | 1.79E-09 |  | 0.019 | 0.030 | 5.29E-01 |  | 0.283 | 0.046 | 1.03E-09 |
| rs35613615 | 3 | 46265243 | C | T | 0.019 | 0.003 | 5.57E-10 |  | 0.006 | 0.032 | 8.48E-01 |  | 0.307 | 0.048 | 1.27E-10 |
| rs35614049 | 3 | 46255476 | C | T | 0.018 | 0.003 | 1.73E-09 |  | 0.017 | 0.030 | 5.65E-01 |  | 0.284 | 0.046 | 8.99E-10 |
| rs35617677 | 3 | 46258740 | T | C | 0.018 | 0.003 | 1.84E-09 |  | 0.019 | 0.030 | 5.35E-01 |  | 0.283 | 0.046 | 1.04E-09 |
| rs35646896 | 3 | 46320120 | T | G | 0.017 | 0.003 | 1.38E-08 |  | 0.029 | 0.032 | 3.67E-01 |  | 0.281 | 0.050 | 1.45E-08 |
| rs35669129 | 3 | 46055250 | A | G | 0.026 | 0.003 | 5.71E-24 |  | 0.010 | 0.027 | 7.20E-01 |  | 0.405 | 0.039 | 1.40E-25 |
| rs35685805 | 3 | 46202778 | C | T | 0.018 | 0.003 | 9.34E-10 |  | 0.025 | 0.030 | 4.12E-01 |  | 0.284 | 0.046 | 7.39E-10 |
| rs35751180 | 3 | 46056173 | T | C | 0.027 | 0.003 | 1.42E-24 |  | 0.007 | 0.027 | 7.87E-01 |  | 0.421 | 0.040 | 2.35E-26 |
| rs35754688 | 3 | 46187706 | T | C | 0.018 | 0.003 | 1.43E-09 |  | 0.027 | 0.030 | 3.72E-01 |  | 0.278 | 0.046 | 1.32E-09 |
| rs35772789 | 3 | 46095104 | G | A | 0.026 | 0.003 | 2.31E-22 |  | 0.000 | 0.027 | 9.98E-01 |  | 0.409 | 0.040 | 2.60E-24 |
| rs35775079 | 3 | 46262111 | T | C | 0.018 | 0.003 | 1.69E-09 |  | 0.019 | 0.030 | 5.32E-01 |  | 0.283 | 0.046 | 9.55E-10 |
| rs35827997 | 3 | 46001720 | G | T | 0.027 | 0.003 | 2.13E-24 |  | 0.010 | 0.026 | 7.02E-01 |  | 0.414 | 0.039 | 5.19E-26 |
| rs35831747 | 3 | 45970391 | A | G | 0.027 | 0.003 | 8.75E-25 |  | 0.005 | 0.027 | 8.63E-01 |  | 0.425 | 0.040 | 1.02E-26 |
| rs35855315 | 3 | 46006239 | G | A | 0.027 | 0.003 | 8.56E-25 |  | 0.011 | 0.027 | 6.72E-01 |  | 0.417 | 0.039 | 2.21E-26 |
| rs35883205 | 3 | 46053968 | G | A | 0.027 | 0.003 | 3.29E-24 |  | 0.009 | 0.027 | 7.29E-01 |  | 0.416 | 0.040 | 7.44E-26 |
| rs35919278 | 3 | 46213594 | G | A | 0.018 | 0.003 | 8.73E-10 |  | 0.024 | 0.030 | 4.32E-01 |  | 0.286 | 0.046 | 6.44E-10 |
| rs35942803 | 3 | 46208783 | C | T | 0.018 | 0.003 | 9.43E-10 |  | 0.024 | 0.030 | 4.32E-01 |  | 0.285 | 0.046 | 7.00E-10 |
| rs36010446 | 3 | 46226725 | A | G | 0.018 | 0.003 | 8.73E-10 |  | 0.023 | 0.030 | 4.50E-01 |  | 0.285 | 0.046 | 6.10E-10 |
| rs36039366 | 3 | 46003537 | A | G | 0.027 | 0.003 | 2.26E-24 |  | 0.01 | 0.026 | 7.19E-01 |  | 0.414 | 0.039 | 5.13E-26 |
| rs36078103 | 3 | 46311782 | T | C | 0.018 | 0.003 | 9.22E-09 |  | 0.029 | 0.032 | 3.63E-01 |  | 0.284 | 0.049 | 9.61E-09 |
| rs36122610 | 3 | 46022833 | A | G | 0.027 | 0.003 | 7.71E-25 |  | 0.008 | 0.027 | 7.52E-01 |  | 0.421 | 0.039 | 1.40E-26 |
| rs3851347 | 3 | 46053778 | G | A | 0.011 | 0.002 | 4.18E-10 |  | 0.000 | 0.017 | 9.99E-01 |  | 0.179 | 0.027 | 6.41E-11 |
| rs41289616 | 3 | 45997263 | C | T | 0.027 | 0.003 | 1.77E-24 |  | 0.010 | 0.027 | 6.96E-01 |  | 0.415 | 0.039 | 4.36E-26 |
| rs41289622 | 3 | 46014545 | G | T | 0.027 | 0.003 | 4.42E-25 |  | 0.008 | 0.027 | 7.71E-01 |  | 0.424 | 0.039 | 7.05E-27 |
| rs41432345 | 3 | 46250374 | C | T | 0.016 | 0.003 | 1.11E-08 |  | 0.028 | 0.029 | 3.42E-01 |  | 0.248 | 0.044 | 1.26E-08 |
| rs4388012 | 3 | 45996501 | G | A | 0.027 | 0.003 | 2.32E-24 |  | 0.009 | 0.026 | 7.27E-01 |  | 0.414 | 0.039 | 5.13E-26 |
| rs4443214 | 3 | 46177864 | C | T | 0.018 | 0.003 | 1.63E-12 |  | 0.013 | 0.027 | 6.26E-01 |  | 0.293 | 0.040 | 4.39E-13 |
| rs4473594 | 3 | 46337356 | A | G | 0.017 | 0.003 | 2.03E-08 |  | 0.029 | 0.032 | 3.76E-01 |  | 0.281 | 0.050 | 2.10E-08 |
| rs4493469 | 3 | 46177992 | C | T | 0.020 | 0.003 | 5.88E-14 |  | 0.003 | 0.028 | 9.16E-01 |  | 0.332 | 0.042 | 5.10E-15 |
| rs4683163 | 3 | 46084372 | C | A | 0.023 | 0.002 | 4.08E-21 |  | 0.000 | 0.025 | 9.94E-01 |  | 0.363 | 0.037 | 6.10E-23 |
| rs4987053 | 3 | 46306700 | C | T | 0.017 | 0.003 | 9.75E-09 |  | 0.044 | 0.032 | 1.69E-01 |  | 0.269 | 0.048 | 2.29E-08 |
| rs55875328 | 3 | 46051702 | A | G | 0.027 | 0.003 | 2.50E-24 |  | 0.012 | 0.027 | 6.45E-01 |  | 0.407 | 0.039 | 7.92E-26 |
| rs56332428 | 3 | 45989873 | T | C | 0.027 | 0.003 | 1.63E-24 |  | 0.009 | 0.026 | 7.29E-01 |  | 0.416 | 0.039 | 3.46E-26 |
| rs61650989 | 3 | 46054156 | T | G | 0.026 | 0.003 | 6.58E-24 |  | 0.009 | 0.027 | 7.22E-01 |  | 0.405 | 0.039 | 1.61E-25 |
| rs67200151 | 3 | 46051774 | A | G | 0.027 | 0.003 | 2.95E-24 |  | 0.009 | 0.027 | 7.26E-01 |  | 0.408 | 0.039 | 6.68E-26 |
| rs6764042 | 3 | 46059139 | G | T | 0.011 | 0.002 | 1.28E-09 |  | 0.000 | 0.017 | 9.87E-01 |  | 0.174 | 0.027 | 2.24E-10 |
| rs6765904 | 3 | 46195881 | G | A | 0.017 | 0.003 | 1.36E-09 |  | 0.029 | 0.03 | 3.25E-01 |  | 0.274 | 0.045 | 1.49E-09 |
| rs67676925 | 3 | 46274259 | C | T | 0.018 | 0.003 | 9.98E-09 |  | 0.014 | 0.031 | 6.46E-01 |  | 0.285 | 0.049 | 4.69E-09 |
| rs6793370 | 3 | 46312847 | G | A | 0.017 | 0.003 | 1.57E-08 |  | 0.040 | 0.032 | 2.05E-01 |  | 0.266 | 0.048 | 3.10E-08 |
| rs67937868 | 3 | 46049827 | T | G | 0.027 | 0.003 | 3.34E-24 |  | 0.011 | 0.027 | 6.93E-01 |  | 0.408 | 0.039 | 8.75E-26 |
| rs6803341 | 3 | 46315657 | G | A | 0.017 | 0.003 | 1.78E-08 |  | 0.041 | 0.032 | 1.95E-01 |  | 0.265 | 0.048 | 3.70E-08 |
| rs71325095 | 3 | 45989921 | T | G | 0.027 | 0.003 | 7.83E-25 |  | 0.011 | 0.027 | 6.70E-01 |  | 0.418 | 0.039 | 2.03E-26 |
| rs71325100 | 3 | 46013832 | T | C | 0.027 | 0.003 | 4.97E-25 |  | 0.008 | 0.027 | 7.53E-01 |  | 0.423 | 0.039 | 8.69E-27 |
| rs71325101 | 3 | 46015569 | C | T | 0.027 | 0.003 | 6.52E-25 |  | 0.011 | 0.027 | 6.82E-01 |  | 0.418 | 0.039 | 1.57E-26 |
| rs71325102 | 3 | 46015933 | C | T | 0.027 | 0.003 | 6.40E-25 |  | 0.013 | 0.026 | 6.29E-01 |  | 0.417 | 0.039 | 1.95E-26 |
| rs71327003 | 3 | 46036521 | T | C | 0.017 | 0.003 | 9.11E-11 |  | 0.009 | 0.027 | 7.47E-01 |  | 0.345 | 0.052 | 2.29E-11 |
| rs71327006 | 3 | 46058908 | A | G | 0.026 | 0.003 | 1.66E-22 |  | 0.000 | 0.027 | 9.98E-01 |  | 0.405 | 0.040 | 1.82E-24 |
| rs71327014 | 3 | 46101466 | T | C | 0.026 | 0.003 | 2.18E-22 |  | 0.001 | 0.027 | 9.76E-01 |  | 0.409 | 0.040 | 2.66E-24 |
| rs71327024 | 3 | 46140073 | T | G | 0.025 | 0.003 | 1.17E-21 |  | 0.000 | 0.027 | 9.98E-01 |  | 0.404 | 0.040 | 1.54E-23 |
| rs71327025 | 3 | 46147504 | G | A | 0.016 | 0.003 | 2.80E-09 |  | 0.000 | 0.027 | 9.96E-01 |  | 0.331 | 0.053 | 5.02E-10 |
| rs71327035 | 3 | 46194528 | C | T | 0.016 | 0.003 | 6.82E-09 |  | 0.032 | 0.029 | 2.76E-01 |  | 0.256 | 0.045 | 9.62E-09 |
| rs71327036 | 3 | 46194589 | A | C | 0.016 | 0.003 | 6.39E-09 |  | 0.032 | 0.030 | 2.89E-01 |  | 0.256 | 0.045 | 8.54E-09 |
| rs71327038 | 3 | 46225995 | C | A | 0.018 | 0.003 | 9.56E-10 |  | 0.023 | 0.030 | 4.40E-01 |  | 0.285 | 0.046 | 6.91E-10 |
| rs71327039 | 3 | 46257296 | C | A | 0.018 | 0.003 | 1.82E-09 |  | 0.019 | 0.030 | 5.37E-01 |  | 0.283 | 0.046 | 1.02E-09 |
| rs71327040 | 3 | 46263208 | T | G | 0.019 | 0.003 | 5.49E-10 |  | 0.006 | 0.032 | 8.51E-01 |  | 0.307 | 0.048 | 1.25E-10 |
| rs71327041 | 3 | 46267582 | T | C | 0.019 | 0.003 | 4.77E-10 |  | 0.008 | 0.032 | 8.01E-01 |  | 0.307 | 0.048 | 1.22E-10 |
| rs71327042 | 3 | 46268013 | C | T | 0.019 | 0.003 | 5.68E-10 |  | 0.006 | 0.032 | 8.51E-01 |  | 0.307 | 0.048 | 1.29E-10 |
| rs71327047 | 3 | 46310840 | C | T | 0.018 | 0.003 | 1.30E-08 |  | 0.029 | 0.032 | 3.66E-01 |  | 0.286 | 0.050 | 1.37E-08 |
| rs71327048 | 3 | 46350056 | C | A | 0.017 | 0.003 | 1.93E-08 |  | 0.033 | 0.032 | 3.10E-01 |  | 0.280 | 0.050 | 2.48E-08 |
| rs71615438 | 3 | 46039868 | G | A | 0.027 | 0.003 | 2.18E-24 |  | 0.009 | 0.027 | 7.25E-01 |  | 0.417 | 0.039 | 4.84E-26 |
| rs72901034 | 3 | 46085321 | G | A | 0.023 | 0.002 | 3.83E-21 |  | 0.001 | 0.025 | 9.73E-01 |  | 0.363 | 0.037 | 6.17E-23 |
| rs74586549 | 3 | 45926043 | T | C | 0.017 | 0.002 | 3.03E-11 |  | 0.005 | 0.026 | 8.36E-01 |  | 0.342 | 0.050 | 5.47E-12 |
| rs75928798 | 3 | 45962603 | G | A | 0.027 | 0.003 | 3.26E-25 |  | 0.007 | 0.027 | 8.02E-01 |  | 0.430 | 0.040 | 4.46E-27 |
| rs7620586 | 3 | 46267737 | G | T | 0.016 | 0.003 | 4.48E-08 |  | 0.012 | 0.031 | 6.97E-01 |  | 0.250 | 0.045 | 2.07E-08 |
| rs7623190 | 3 | 46068325 | C | T | 0.011 | 0.002 | 1.27E-09 |  | 0.000 | 0.017 | 1.00E+00 |  | 0.175 | 0.028 | 2.14E-10 |
| rs76281521 | 3 | 46049764 | A | G | 0.027 | 0.003 | 5.79E-25 |  | 0.013 | 0.027 | 6.32E-01 |  | 0.423 | 0.040 | 1.73E-26 |
| rs7631551 | 3 | 46186310 | A | C | 0.017 | 0.003 | 3.37E-09 |  | 0.030 | 0.030 | 3.09E-01 |  | 0.263 | 0.045 | 4.07E-09 |
| rs7631853 | 3 | 46306474 | C | T | 0.018 | 0.003 | 9.44E-09 |  | 0.044 | 0.032 | 1.66E-01 |  | 0.269 | 0.048 | 2.25E-08 |
| rs7642229 | 3 | 46090557 | G | A | 0.023 | 0.002 | 4.96E-21 |  | 0.001 | 0.025 | 9.58E-01 |  | 0.363 | 0.037 | 8.60E-23 |
| rs7642320 | 3 | 46090622 | G | A | 0.023 | 0.002 | 4.08E-21 |  | 0.001 | 0.025 | 9.68E-01 |  | 0.364 | 0.037 | 6.70E-23 |
| rs7649905 | 3 | 46316005 | C | T | 0.017 | 0.003 | 1.82E-08 |  | 0.041 | 0.032 | 1.95E-01 |  | 0.265 | 0.048 | 3.79E-08 |
| rs7651539 | 3 | 46268079 | T | C | 0.016 | 0.003 | 4.50E-08 |  | 0.012 | 0.031 | 6.97E-01 |  | 0.250 | 0.045 | 2.07E-08 |
| rs7652478 | 3 | 46090477 | A | G | 0.023 | 0.002 | 4.09E-21 |  | 0.002 | 0.025 | 9.51E-01 |  | 0.363 | 0.037 | 7.14E-23 |
| rs7653372 | 3 | 45927759 | C | T | 0.016 | 0.002 | 7.71E-11 |  | 0.009 | 0.026 | 7.38E-01 |  | 0.332 | 0.050 | 1.96E-11 |
| rs76597151 | 3 | 46001227 | A | G | 0.027 | 0.003 | 2.19E-24 |  | 0.010 | 0.026 | 7.18E-01 |  | 0.414 | 0.039 | 5.00E-26 |
| rs76836071 | 3 | 46344999 | T | C | 0.017 | 0.003 | 2.29E-08 |  | 0.032 | 0.032 | 3.25E-01 |  | 0.279 | 0.050 | 2.82E-08 |
| rs77902290 | 3 | 45976662 | T | C | 0.027 | 0.003 | 8.99E-25 |  | 0.004 | 0.027 | 8.77E-01 |  | 0.425 | 0.040 | 9.87E-27 |
| rs78124692 | 3 | 46202237 | T | C | 0.018 | 0.003 | 9.27E-10 |  | 0.025 | 0.030 | 4.12E-01 |  | 0.284 | 0.046 | 7.33E-10 |
| rs78919248 | 3 | 46279150 | T | C | 0.027 | 0.004 | 6.65E-14 |  | 0.026 | 0.038 | 4.97E-01 |  | 0.442 | 0.058 | 2.23E-14 |
| rs79948053 | 3 | 46211060 | A | G | 0.018 | 0.003 | 9.26E-10 |  | 0.024 | 0.030 | 4.28E-01 |  | 0.284 | 0.046 | 6.94E-10 |
| rs80007414 | 3 | 46202238 | G | A | 0.018 | 0.003 | 9.30E-10 |  | 0.025 | 0.030 | 4.12E-01 |  | 0.284 | 0.046 | 7.36E-10 |
| rs9810242 | 3 | 46313334 | G | A | 0.017 | 0.003 | 2.02E-08 |  | 0.041 | 0.032 | 1.95E-01 |  | 0.263 | 0.048 | 4.22E-08 |
| rs9838450 | 3 | 46123333 | C | A | 0.018 | 0.002 | 9.71E-16 |  | 0.004 | 0.023 | 8.68E-01 |  | 0.301 | 0.036 | 6.81E-17 |
| rs9845542 | 3 | 46111869 | A | G | 0.018 | 0.002 | 6.14E-15 |  | 0.002 | 0.023 | 9.20E-01 |  | 0.288 | 0.035 | 4.30E-16 |
| rs9872023 | 3 | 46322713 | T | C | 0.017 | 0.003 | 3.56E-08 |  | 0.032 | 0.032 | 3.13E-01 |  | 0.267 | 0.049 | 4.66E-08 |
| rs1859336 | 12 | 113367309 | T | C | 0.010 | 0.002 | 1.02E-09 |  | 0.003 | 0.016 | 8.78E-01 |  | 0.178 | 0.028 | 2.28E-10 |
| rs2384071 | 12 | 113367343 | G | A | 0.010 | 0.002 | 1.26E-09 |  | 0.001 | 0.016 | 9.68E-01 |  | 0.177 | 0.028 | 2.30E-10 |
| rs77534576 | 17 | 47940666 | T | C | 0.028 | 0.005 | 2.20E-09 |  | 0.020 | 0.049 | 6.81E-01 |  | 0.460 | 0.075 | 8.53E-10 |
| rs11085727 | 19 | 10466123 | T | C | 0.011 | 0.002 | 2.75E-10 |  | 0.041 | 0.017 | 1.74E-02 |  | 0.173 | 0.029 | 3.74E-09 |
| rs12610495 | 19 | 4717672 | G | A | 0.012 | 0.002 | 1.65E-11 |  | 0.026 | 0.017 | 1.36E-01 |  | 0.245 | 0.037 | 4.09E-11 |
| rs2109069 | 19 | 4719443 | A | G | 0.016 | 0.002 | 5.64E-20 |  | 0.024 | 0.017 | 1.49E-01 |  | 0.257 | 0.028 | 6.12E-20 |
| rs2277732 | 19 | 4723670 | A | C | 0.016 | 0.002 | 3.70E-19 |  | 0.018 | 0.017 | 2.93E-01 |  | 0.262 | 0.029 | 1.51E-19 |
| rs2277735 | 19 | 4700187 | G | A | -0.010 | 0.002 | 4.73E-08 |  | -0.012 | 0.018 | 5.13E-01 |  | -0.161 | 0.029 | 3.42E-08 |
| rs2304256 | 19 | 10475652 | A | C | 0.011 | 0.002 | 1.61E-09 |  | 0.041 | 0.017 | 1.84E-02 |  | 0.164 | 0.029 | 2.03E-08 |
| rs281422 | 19 | 10436355 | C | T | -0.012 | 0.002 | 9.49E-09 |  | -0.053 | 0.021 | 1.03E-02 |  | -0.181 | 0.035 | 1.61E-07 |
| rs281427 | 19 | 10429120 | C | T | -0.012 | 0.002 | 9.42E-09 |  | -0.054 | 0.021 | 9.46E-03 |  | -0.181 | 0.035 | 1.68E-07 |
| rs34725611 | 19 | 10477067 | G | A | 0.011 | 0.002 | 2.68E-09 |  | 0.041 | 0.017 | 1.88E-02 |  | 0.162 | 0.029 | 3.29E-08 |
| rs7255545 | 19 | 4724734 | A | G | 0.011 | 0.002 | 1.22E-10 |  | 0.021 | 0.016 | 2.08E-01 |  | 0.228 | 0.036 | 2.02E-10 |
| rs7256695 | 19 | 4703224 | T | C | 0.009 | 0.002 | 1.47E-08 |  | 0.021 | 0.016 | 1.98E-01 |  | 0.200 | 0.036 | 2.97E-08 |
| rs8101195 | 19 | 10423815 | A | G | -0.012 | 0.002 | 1.12E-08 |  | -0.051 | 0.021 | 1.33E-02 |  | -0.182 | 0.035 | 1.64E-07 |
| rs8101473 | 19 | 10423338 | T | C | -0.012 | 0.002 | 1.18E-08 |  | -0.052 | 0.021 | 1.26E-02 |  | -0.181 | 0.035 | 1.78E-07 |
| rs1051393 | 21 | 34614255 | G | T | 0.010 | 0.002 | 1.46E-08 |  | 0.001 | 0.017 | 9.60E-01 |  | 0.169 | 0.029 | 3.40E-09 |
| rs12053666 | 21 | 34618439 | A | G | 0.010 | 0.002 | 2.03E-08 |  | 0.000 | 0.017 | 9.79E-01 |  | 0.171 | 0.029 | 4.66E-09 |
| rs12482556 | 21 | 34602934 | C | T | 0.010 | 0.002 | 3.85E-09 |  | 0.002 | 0.017 | 8.90E-01 |  | 0.175 | 0.029 | 9.36E-10 |
| rs12627039 | 21 | 34600508 | T | G | 0.010 | 0.002 | 2.06E-08 |  | 0.000 | 0.018 | 9.93E-01 |  | 0.171 | 0.029 | 4.56E-09 |
| rs13050728 | 21 | 34615210 | C | T | -0.012 | 0.002 | 1.68E-11 |  | -0.002 | 0.017 | 9.09E-01 |  | -0.200 | 0.029 | 2.44E-12 |
| rs17860115 | 21 | 34602305 | A | C | 0.010 | 0.002 | 2.68E-09 |  | 0.001 | 0.017 | 9.58E-01 |  | 0.177 | 0.029 | 5.37E-10 |
| rs17860169 | 21 | 34613301 | G | A | 0.010 | 0.002 | 3.25E-09 |  | 0.002 | 0.017 | 9.25E-01 |  | 0.176 | 0.029 | 7.16E-10 |
| rs2073361 | 21 | 34619445 | G | A | -0.012 | 0.002 | 2.00E-11 |  | -0.003 | 0.017 | 8.56E-01 |  | -0.199 | 0.029 | 3.40E-12 |
| rs2236756 | 21 | 34616923 | A | C | -0.011 | 0.002 | 4.36E-11 |  | -0.002 | 0.017 | 9.12E-01 |  | -0.196 | 0.029 | 6.82E-12 |
| rs2236757 | 21 | 34624917 | G | A | -0.011 | 0.002 | 1.65E-10 |  | 0.000 | 0.018 | 9.95E-01 |  | -0.195 | 0.029 | 2.34E-11 |
| rs2252639 | 21 | 34617729 | G | A | -0.011 | 0.002 | 2.78E-11 |  | -0.002 | 0.017 | 8.84E-01 |  | -0.198 | 0.029 | 4.51E-12 |
| rs2284551 | 21 | 34618313 | G | A | -0.011 | 0.002 | 1.73E-10 |  | 0.000 | 0.018 | 9.85E-01 |  | -0.195 | 0.029 | 2.54E-11 |
| rs2300370 | 21 | 34604557 | A | G | 0.010 | 0.002 | 2.52E-09 |  | 0.002 | 0.017 | 8.99E-01 |  | 0.177 | 0.029 | 5.77E-10 |
| rs2834154 | 21 | 34606634 | C | A | 0.010 | 0.002 | 5.22E-09 |  | 0.001 | 0.017 | 9.34E-01 |  | 0.174 | 0.029 | 1.17E-09 |
| rs2834161 | 21 | 34620207 | T | C | -0.011 | 0.002 | 3.58E-11 |  | -0.002 | 0.017 | 8.95E-01 |  | -0.197 | 0.029 | 5.75E-12 |
| rs2834163 | 21 | 34620451 | A | G | -0.012 | 0.002 | 1.99E-11 |  | -0.003 | 0.017 | 8.67E-01 |  | -0.199 | 0.029 | 3.28E-12 |
| rs6517153 | 21 | 34607436 | A | G | 0.010 | 0.002 | 3.63E-09 |  | 0.002 | 0.017 | 8.99E-01 |  | 0.175 | 0.029 | 8.58E-10 |
| rs9636867 | 21 | 34609944 | G | A | 0.010 | 0.002 | 2.40E-09 |  | 0.003 | 0.017 | 8.68E-01 |  | 0.177 | 0.029 | 5.90E-10 |
| rs9976829 | 21 | 34614834 | A | G | -0.012 | 0.002 | 2.46E-11 |  | -0.002 | 0.017 | 9.23E-01 |  | -0.199 | 0.029 | 3.56E-12 |
| SNP, single nucleotide polymorphism; A1, effect allele; A2, reference allele. | | | | | | | | | | | | | | | |

**Table S5.** Summary of significant SMR associations involving novel SNPs associated with cross-trait OA and COVID-19 outcomes.

| **Gene** | **CHR** | **BP** | **Top SNP** | **Top SNP position** | **A1** | **A2** | **Beta_mtag_** | ***P*_mtag_** | **Beta_SMR_** | ***P*_SMR_** |
| --- | --- | --- | --- | --- | --- | --- | --- | --- | --- | --- |
| OA and critical COVID-19 (eQTL data from lung tissue) | | | | | | | | | | |
| *TCF19* | 6 | 31126319 | rs143334143 | 31121426 | A | G | 0.019 | 1.02E-10 | 0.066 | 2.96E-06 |
| *XXbac-BPG299F13.17* | 6 | 31162977 | rs143334143 | 31121426 | A | G | 0.019 | 1.02E-10 | 0.022 | 1.05E-08 |
| *HLA-C* | 6 | 31236526 | rs143334143 | 31121426 | A | G | 0.019 | 1.02E-10 | 0.039 | 4.90E-06 |
| *DLX3* | 17 | 48067369 | rs77534576 | 47940666 | T | C | 0.028 | 2.20E-09 | 0.031 | 1.67E-05 |
| *DPP9* | 19 | 4675236 | rs2277732 | 4723670 | A | C | 0.016 | 3.70E-19 | -0.08 | 2.99E-07 |
| OA and critical COVID-19 (eQTL data from blood) | | | | | | | | | | |
| *FLT1P1* | 3 | 46183850 | rs13081151 | 46055716 | A | G | 0.027 | 5.95E-24 | -0.087 | 4.05E-08 |
| *TCF19* | 6 | 31126319 | rs143334143 | 31121426 | A | G | 0.019 | 1.02E-10 | 0.061 | 5.38E-08 |
| *HCG27* | 6 | 31165537 | rs143334143 | 31121426 | A | G | 0.019 | 1.02E-10 | 0.083 | 1.65E-06 |
| *HLA-C* | 6 | 31236526 | rs143334143 | 31121426 | A | G | 0.019 | 1.02E-10 | 0.050 | 6.91E-07 |
| *XXbac-BPG181B23.7* | 6 | 31362066 | rs143334143 | 31121426 | A | G | 0.019 | 1.02E-10 | -0.045 | 5.90E-06 |
| *OAS1* | 12 | 113344582 | rs10774671 | 113357193 | A | G | 0.011 | 1.69E-10 | -0.066 | 1.24E-05 |
| *OAS3* | 12 | 113376157 | rs7955267 | 113379039 | T | C | 0.010 | 6.34E-10 | 0.070 | 1.53E-05 |
| *TYK2* | 19 | 10461209 | rs11085727 | 10466123 | T | C | 0.011 | 2.75E-10 | 0.096 | 6.81E-07 |
| OA and COVID-19 hospitalization (eQTL data from lung tissue) | | | | | | | | | | |
| *DPP9* | 19 | 4675236 | rs2277732 | 4723670 | A | C | 0.010 | 1.03E-13 | -0.053 | 1.71E-06 |
| OA and COVID-19 hospitalization (eQTL data from blood) | | | | | | | | | | |
| *ADAM15* | 1 | 155023042 | rs6675468 | 155040654 | T | C | -0.014 | 1.82E-08 | 0.040 | 1.79E-06 |
| *FLT1P1* | 3 | 46183850 | rs13081151 | 46055716 | A | G | 0.024 | 2.56E-32 | -0.077 | 1.04E-08 |
| *CCR1* | 3 | 46243200 | rs4682799 | 45993606 | C | T | 0.007 | 6.71E-09 | -0.042 | 1.01E-05 |
| *ABO* | 9 | 136125788 | rs495828 | 136154867 | G | T | -0.008 | 4.94E-08 | -0.012 | 7.43E-07 |
| *OAS1* | 12 | 113344582 | rs10774671 | 113357193 | A | G | 0.008 | 1.54E-08 | -0.046 | 3.89E-05 |
| *OAS3* | 12 | 113376157 | rs4766664 | 113362997 | G | T | 0.007 | 3.25E-08 | 0.049 | 6.42E-05 |
| *TYK2* | 19 | 10461209 | rs11085727 | 10466123 | T | C | 0.008 | 1.42E-08 | 0.068 | 3.56E-06 |
| *IFNAR2* | 21 | 34602206 | rs1131964 | 34610487 | C | T | 0.007 | 1.82E-08 | -0.076 | 8.67E-05 |
| OA, osteoarthritis; SMR, Summary-data-based Mendelian randomization; SNP: single nucleotide polymorphism; CHR, chromosome number of the SNP; BP, base pair position of the SNP; A1, effect allele of the SNP; A2, the other allele of the SNP. | | | | | | | | | | |

**Table S6.** 34 genome-wide significant independent loci for osteoarthritis.

| **SNP** | **Chr** | **Position** | **A1** | **A2** | **EAF** | **Beta** | **SE** | ***P*** |
| --- | --- | --- | --- | --- | --- | --- | --- | --- |
| rs1886647 | 1 | 85354563 | A | G | 0.280 | -0.084 | 0.018 | 3.43E-06 |
| rs56075670 | 1 | 18418325 | A | G | 0.013 | -0.356 | 0.081 | 4.28E-06 |
| rs12731575 | 1 | 103343274 | C | T | 0.053 | -0.166 | 0.037 | 5.15E-06 |
| rs2820436 | 1 | 219640680 | C | A | 0.657 | -0.075 | 0.017 | 6.45E-06 |
| rs4233567 | 2 | 144272376 | T | C | 0.364 | -0.082 | 0.017 | 1.26E-06 |
| rs6546174 | 2 | 65935393 | T | C | 0.532 | -0.087 | 0.018 | 1.81E-06 |
| rs4380275 | 2 | 773278 | T | C | 0.648 | 0.074 | 0.017 | 7.95E-06 |
| rs11686353 | 2 | 236561934 | C | A | 0.263 | -0.082 | 0.019 | 8.56E-06 |
| rs112354755 | 3 | 162868729 | T | C | 0.059 | -0.173 | 0.035 | 6.41E-07 |
| rs73080980 | 3 | 50196533 | T | C | 0.205 | -0.095 | 0.020 | 1.99E-06 |
| rs76372390 | 3 | 186047902 | A | G | 0.005 | 0.602 | 0.129 | 9.99E-06 |
| rs76590116 | 4 | 67170632 | T | C | 0.013 | 0.352 | 0.065 | 1.22E-07 |
| rs148091331 | 5 | 138848661 | T | C | 0.009 | 0.417 | 0.083 | 1.36E-06 |
| rs78115154 | 5 | 152899101 | C | T | 0.038 | -0.197 | 0.044 | 3.97E-06 |
| rs62374753 | 5 | 124768796 | A | G | 0.029 | 0.211 | 0.046 | 5.99E-06 |
| rs7714497 | 5 | 112006948 | G | A | 0.184 | -0.092 | 0.021 | 9.07E-06 |
| rs12193876 | 6 | 89260265 | T | C | 0.176 | -0.100 | 0.021 | 2.54E-06 |
| rs9342489 | 6 | 66344801 | G | A | 0.031 | -0.221 | 0.048 | 2.92E-06 |
| rs3851225 | 6 | 111825132 | G | A | 0.575 | -0.071 | 0.016 | 8.49E-06 |
| rs201708019 | 7 | 136519898 | C | T | 0.701 | -0.086 | 0.019 | 4.14E-06 |
| rs6977416 | 7 | 150542711 | A | G | 0.334 | -0.076 | 0.017 | 7.84E-06 |
| rs72718147 | 9 | 25036933 | A | G | 0.155 | -0.107 | 0.024 | 5.71E-06 |
| rs116882138 | 9 | 27313557 | A | G | 0.017 | 0.262 | 0.057 | 6.05E-06 |
| rs75944996 | 9 | 72409180 | G | A | 0.046 | -0.175 | 0.040 | 6.63E-06 |
| rs140527764 | 9 | 112864137 | A | C | 0.008 | 0.412 | 0.089 | 7.53E-06 |
| rs181549559 | 10 | 93397289 | T | C | 0.008 | 0.459 | 0.098 | 7.21E-06 |
| rs17712916 | 12 | 64607264 | A | G | 0.057 | 0.149 | 0.033 | 7.29E-06 |
| rs11620787 | 14 | 35096198 | A | G | 0.180 | 0.097 | 0.020 | 1.72E-06 |
| rs8097350 | 18 | 76262424 | T | C | 0.454 | -0.079 | 0.017 | 4.69E-06 |
| rs80142449 | 19 | 41712989 | A | G | 0.019 | 0.260 | 0.057 | 9.30E-06 |
| rs143383 | 20 | 34025983 | G | A | 0.361 | -0.084 | 0.017 | 3.53E-07 |
| rs1044369 | 20 | 44987318 | G | C | 0.313 | -0.077 | 0.017 | 7.23E-06 |
| rs143983158 | 20 | 21616475 | A | G | 0.024 | -0.277 | 0.064 | 7.29E-06 |
| rs2231495 | 22 | 17669306 | C | T | 0.326 | 0.078 | 0.017 | 3.01E-06 |
| SNP, single nucleotide polymorphism; A1, effect allele; A2, reference allele; EAF, effect allele frequency. | | | | | | | | |

**Table S7.** The genome-wide significant independent loci for obesity-related traits.

| **SNP** | **Chr** | **Position** | **A1** | **A2** | **Trait** | **EAF** | **Beta** | **SE** | ***P*** |
| --- | --- | --- | --- | --- | --- | --- | --- | --- | --- |
| rs2820436 | 1 | 219640680 | C | A | Hip circumference | 0.657 | -0.030 | 0.003 | 3.84E-32 |
| rs73080980 | 3 | 50196533 | T | C | BMI | 0.201 | -0.021 | 0.003 | 2.83E-12 |
| rs6977416 | 7 | 150542711 | A | G | Body fat percentage | 0.331 | -0.011 | 0.002 | 3.82E-08 |
| rs143383 | 20 | 34025983 | G | A | Weight | 0.360 | 0.030 | 0.002 | 2.71E-42 |
| SNP, single nucleotide polymorphism; A1, effect allele; A2, reference allele; EAF, effect allele frequency. BMI, body mass index. | | | | | | | | | |

**Table S8.** Results of Mendelian randomization analyses evaluating causal relationships between osteoarthritis and COVID-19 outcomes after removing obesity-related SNPs.

| **Outcomes** | **N.SNPs** | **Methods** | **OR** | **95% CI** | ***P* value** |
| --- | --- | --- | --- | --- | --- |
| Critical COVID-19 | 24 | Inverse variance weighted | 1.19 | 1.01, 1.40 | 0.043 |
|  |  | MR Egger | 1.76 | 1.20, 2.59 | 0.009 |
|  |  | Weighted median | 1.24 | 1.00, 1.54 | 0.051 |
|  |  | Weighted mode | 1.51 | 0.98, 2.31 | 0.073 |
| COVID-19 hospitalization | 22 | Inverse variance weighted | 1.07 | 0.96, 1.20 | 0.223 |
|  |  | MR Egger | 1.18 | 0.89, 1.56 | 0.264 |
|  |  | Weighted median | 1.03 | 0.89, 1.20 | 0.690 |
|  |  | Weighted mode | 0.97 | 0.75, 1.26 | 0.805 |
| COVID-19 infection | 22 | Inverse variance weighted | 1.05 | 0.99, 1.11 | 0.089 |
|  |  | MR Egger | 1.09 | 0.94, 1.25 | 0.267 |
|  |  | Weighted median | 1.03 | 0.96, 1.11 | 0.398 |
|  |  | Weighted mode | 1.03 | 0.89, 1.18 | 0.725 |
| N.SNPs, number of single nucleotide polymorphisms; OR, odds ratio; CI, confidence interval. | | | | | |

**
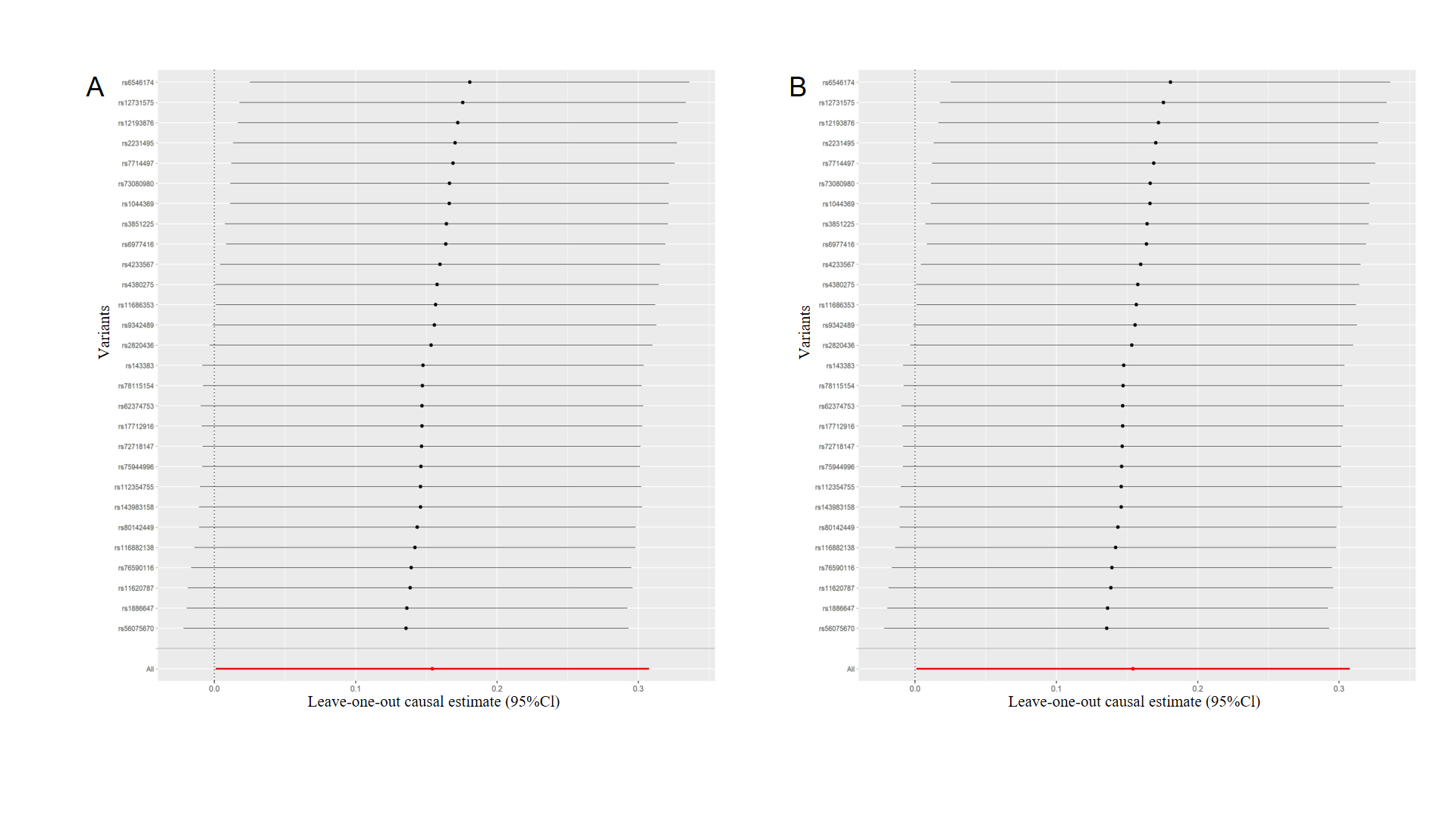
**

**Figure S1.** Leave-one-out analysis related to the osteoarthritis genetic instruments with respect to critical COVID-19 (A) and COVID-19 hospitalization (B).


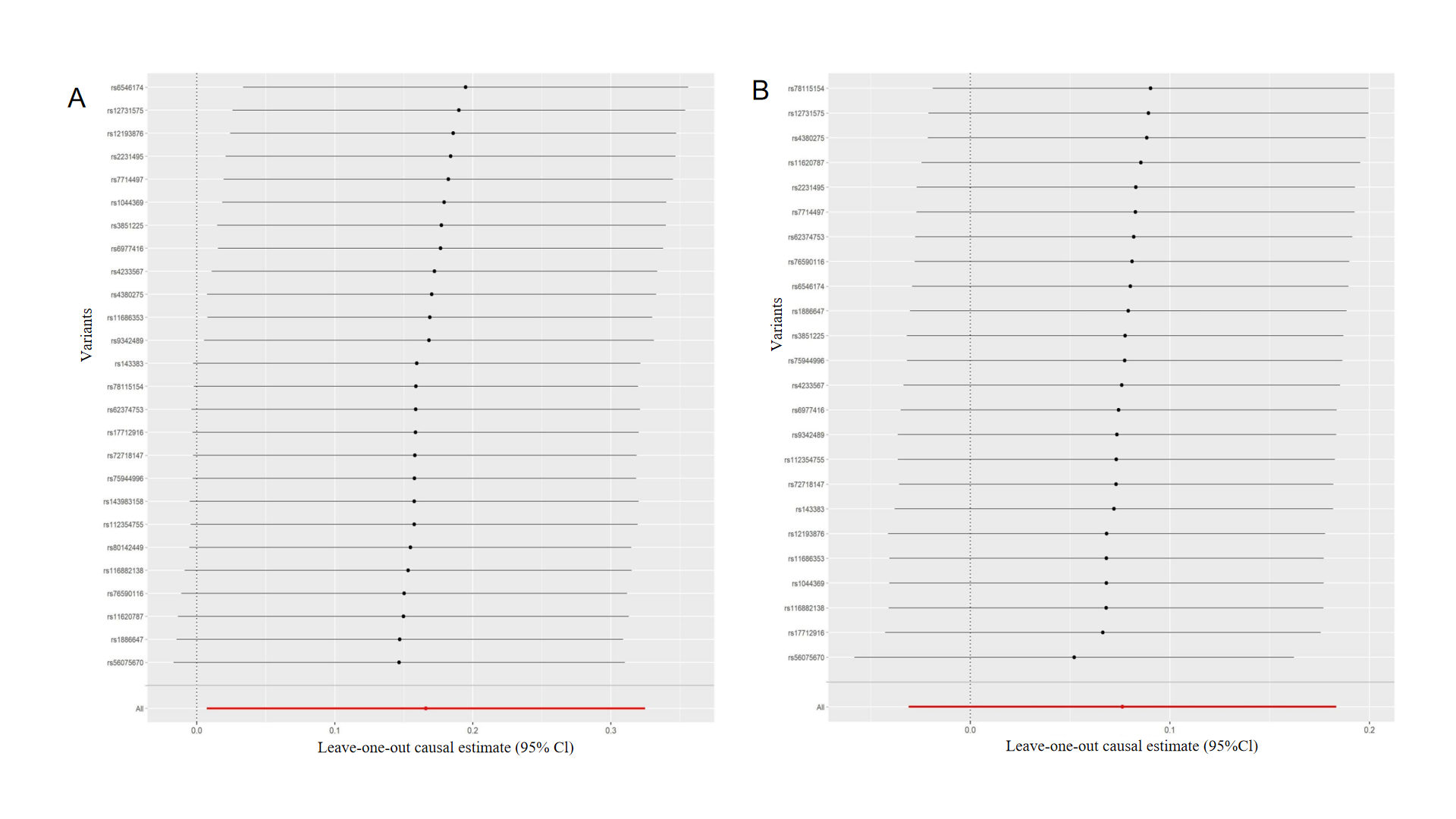
**Figure S2.** Leave-one-out analysis related to the osteoarthritis genetic instruments with respect to critical COVID-19 (A) and COVID-19 hospitalization (B). (obesity-related SNPs excluded).
